# Recent Advances in Electrochemical Sensing of Hydrogen Peroxide (H_2_O_2_) Released from Cancer Cells

**DOI:** 10.3390/nano12091475

**Published:** 2022-04-26

**Authors:** Touqeer Ahmad, Ayesha Iqbal, Sobia Ahsan Halim, Jalal Uddin, Ajmal Khan, Sami El Deeb, Ahmed Al-Harrasi

**Affiliations:** 1 Natural and Medical Sciences Research Center, University of Nizwa, P.O. Box 33, Birkat Al Mauz, Nizwa 616, Oman; touqeernano@hotmail.com (T.A.); sobia_halim@unizwa.edu.om (S.A.H.); 2 Division of Pharmacy Practice and Policy, School of Pharmacy, University of Nottingham, Nottingham NG7 2RD, UK; ayesharph@hotmail.com; 3 Department of Pharmaceutical Chemistry, College of Pharmacy, King Khalid University, Abha 62529, Saudi Arabia; jalaluddinamin@gmail.com; 4 Institute of Medicinal and Pharmaceutical Chemistry, Technische Universitaet Braunschweig, 38106 Braunschweig, Germany

**Keywords:** analytical methods, biosensors, carbon materials, electrochemical sensing, H_2_O_2_, nanomaterial

## Abstract

Cancer is by far the most common cause of death worldwide. There are more than 200 types of cancer known hitherto depending upon the origin and type. Early diagnosis of cancer provides better disease prognosis and the best chance for a cure. This fact prompts world-leading scientists and clinicians to develop techniques for the early detection of cancer. Thus, less morbidity and lower mortality rates are envisioned. The latest advancements in the diagnosis of cancer utilizing nanotechnology have manifested encouraging results. Cancerous cells are well known for their substantial amounts of hydrogen peroxide (H_2_O_2_). The common methods for the detection of H_2_O_2_ include colorimetry, titration, chromatography, spectrophotometry, fluorimetry, and chemiluminescence. These methods commonly lack selectivity, sensitivity, and reproducibility and have prolonged analytical time. New biosensors are reported to circumvent these obstacles. The production of detectable amounts of H_2_O_2_ by cancerous cells has promoted the use of bio- and electrochemical sensors because of their high sensitivity, selectivity, robustness, and miniaturized point-of-care cancer diagnostics. Thus, this review will emphasize the principles, analytical parameters, advantages, and disadvantages of the latest electrochemical biosensors in the detection of H_2_O_2_. It will provide a summary of the latest technological advancements of biosensors based on potentiometric, impedimetric, amperometric, and voltammetric H_2_O_2_ detection. Moreover, it will critically describe the classification of biosensors based on the material, nature, conjugation, and carbon-nanocomposite electrodes for rapid and effective detection of H_2_O_2_, which can be useful in the early detection of cancerous cells.

## 1. Introduction

Biosensors are simple devices that are small and are generally used in the field of medicine, pharmaceutical industries, environmental technology, and food industry. They are used for the measurement of many biological and chemical substances [1]. Owing to the advancement of science and technology, the research involved in biosensors has successfully made the biosensing devices small and efficient [2]. The use of the latest novel techniques and availability of a new biomaterial have made the biosensors efficient and have extended their use in multiple industries such as pharmaceutical, environmental, agriculture, and industrial laboratories [3]. Biosensors are of many types, and electrobiochemical biosensors have been commonly used for over 20 years in the field of diagnostics to detect biochemicals like glucose, lactate, cholesterol, urea, creatinine, DNA, antigens, antibodies, and cancer markers [4]. Electrobiochemical biosensors are also useful in the analysis of food materials and drinks and are extensively utilized in environmental and pharmaceutical laboratories [5,6]. Cancer is one of the most fatal diseases, and every year, more than 10 million new cases and 6 million deaths are reported worldwide [7,8]. Cancer is linked with high rates of morbidities and mortalities and more than 8.7 million deaths worldwide in 2015 [9]. The cancer incidence in high- and low-income countries is similar to the trend of increase in lower–middle-income countries because of the increase in risk factors associated with cancer [10]. In the United States of America (USA), cancer is the second leading cause of mortality, with heart disease being the first, and in a study published in 2017, it was estimated that more than 0.6 million people will die annually from cancer [8]. The survival rate of cancer patients drastically increases if the cancer is detected in earlier stages. The appearance of alarming systems in cancer patients is usually after cancer has spread in the body to multiple locations or has metastasized in different locations and organs, which characterizes an advanced stage of cancer. Most of the people are diagnosed with cancer at an advanced stage, which causes a high risk for mortality. To have a better disease prognosis, it is imperative that new research should focus on the early detection of cancerous cells in the body.

The use of biosensing devices, which have been designed to detect biochemicals, holds vast potential in the early diagnosis of cancers. Biosensors work by detecting a biological moiety or analyte and then converting it into an electrical signal, which can be detected and analyzed by the biosensor device. Many cancerous cells release specific chemicals called biomarkers, which can be detected using a biosensor device. The specific biomarker levels can also help in analyzing the effectiveness of anticancer therapy. The use of biosensor devices is a promising technique, which can help in early and accurate detection, imaging of cancerous cells, monitoring of angiogenesis, detection of proliferation, and tracking of metastatic changes and the efficacy of anticancer therapeutic regimens [11]. The latest research in biosensor devices using the latest techniques, such as nanotechnology-empowered diagnostics, can help in the identification of specific cancer biomarkers, which can help in the detection of cancer, disease progression, disease remission, and further proliferation. The biomarkers of cancer can be overexpressed proteins, surface antigens, active or inactive metabolites, miRNA, or the cancerous cells themselves. Many biosensors are excellent for use as an effective analytical device because of their capability to detect specific cancer biomarkers due to their highly sensitive, selective, robust, and miniaturized point-of-care cancer diagnostic capability [12]. Hydrogen peroxide (H_2_O_2_) is normally present inside the body and is vital in initiating and performing many important physiological processes. H_2_O_2_ is a by-product of respiratory chain and enzymes oxidases (glucose oxidase, cholesterol oxidase, glutamate oxidase, etc.) [13,14,15,16,17]. Hydrogen peroxide is a reactive oxygen species (ROS), which helps in regulating normal body functions such as cell growth, activation of the immune system, and programmed apoptotic changes [13,14,15,16,17]. The body system normally functions in homeostasis, and an increased level of H_2_O_2_ due to increased production can cause harm to the body. Increased levels of H_2_O_2_ can cause damage to normal cells [18], increase inflammatory responses [19], and cause cancer [20]. H_2_O_2_ regulates cancer cell characteristics, including invasion, proliferation, migration, apoptosis, and angiogenesis. Oxidative stress is associated with high levels of ROS, common in many types of cancerous cells. H_2_O_2_ has a specific role as a second messenger in pro-tumorigenic signaling pathways of cancerous cells [21,22]. GPX2 regulates cancer progression by regulating the hydrogen peroxide level in the cells, so when the level of H_2_O_2_ is downregulated to a normal level and the oxidative stress is relieved, it can help in dysregulating cancer cell homeostasis [23]. H_2_O_2_ has recently been a prime focus of research because of its high biological significance. When studied within living systems, it is noteworthy to check the concentration of H_2_O_2_ in mammals. The cellular compartment concentration has to be in the physiological range of 1 nM to 0.5 µM [24,25]. The latest research in the field of cancer diagnosis via biomarker-based techniques has been evaluated as successful because the process ensures high-precision, reliable, and sensitive data. The processes of biomarker-based cancer diagnostic are simple, which makes it a popular choice. Recent studies are focusing on profiling the cell functions with the efflux of endogenous H_2_O_2_ as potential biomarkers for diagnosing various cancers by measuring them using conventional biological assays [26]. However, before moving to the practical implications of using H_2_O_2_ as a potential biomarker for cancer diagnostics in living systems, detection of increased oxidative stress, prediction of neurodegenerative diseases, and detection of tumor growth inside living organisms, it is imperative to develop methods and techniques that can precisely detect and measure the level of H_2_O_2_ inside the cellular compartments [27].

The current problem with detecting H_2_O_2_ in cellular compartments is its low concentration in the body, as well as reactivity, which makes it difficult to separate its normal physiological concentration in a healthy organism from the concentration in a diseased or high-risk state. Therefore, scientists and researchers are focusing on developing sensors that can detect and quantify H_2_O_2_ in different systems and physiological conditions. Currently, many analytical techniques such as titrimetry, spectrophotometry, chemiluminescence, chromatography, fluorescence, and phosphorescence are measuring and determining the concentration of H_2_O_2_ in cellular compartments to develop consistent, precise, sensitive, fast, efficient, and low-cost methods. Currently, many methods investigated based on these analytical techniques have methodological disadvantages such as small sensitivity and selectivity, time-consuming and complex process, susceptibility to interference, and high-cost running instruments [28]. Alternatively, the advantages of “electrochemical sensors” are that they are highly sensitive and selective, reliable, quick, less costly, simple, and practical, and therefore, they are an optimum solution for exact and sensitive H_2_O_2_ detection [29]. The advancements of science and the latest research are looking for potential solutions to detect cancer at early stages and provide individualized therapeutic regimens. However, these techniques and methods still have many restrictions and limitations in the context of clinical examinations, histopathological analysis, imaging mammography, and chemotherapeutic adverse drug effects, as well as their high running costs [30,31]. In the detection of breast cancer, the patients are first exposed to a high amount of radiation in mammography because of its inadequate test sensitivity. Detection and disease progression are usually confirmed using a biopsy, an invasive procedure used to conduct histopathology of the disease [32]. These commonly used procedures are highly risky, uncomfortable, invasive, and costly. Therefore, the latest research is currently focused on developing noninvasive, inexpensive, highly selective screening, diagnostic, and therapeutic approaches to improve the disease prognosis. Several reviews regarding the importance of the carbon nanomaterial in the electrochemical sensing of H_2_O_2_ have been published. Out of those reviews, Yang et al. and Wang et al. conducted their reviews by comparing different nanomaterials for the creation of electrochemical biosensors and their applications for the detection of biomolecules [33,34]. Other materials have also been used in creating biosensors such as graphene electrochemical biosensors [35,36,37] and chemical sensors [38,39], which have been reviewed by Kuila and coworkers. Depending on the aim of the existing reviews, they provide an overview of biosensors that have been used to detect biological analytes. Similarly, Ping and coworkers [40] reviewed the strengths, advantages, and existing applications of 2D graphene-based aptasensors, whereas Chen and coworkers [13] focused their review on the carbon nanomaterial and transition metals in the electrocatalytic reduction of H_2_O_2_ in different samples. Similarly, Zhang et al. aimed to review the role of carbon materials in improving the sensitivity of H_2_O_2_ biosensors [41]. Regardless of these existing review articles, a comprehensive overview on the carbon-based nanomaterials and their composite with metal NPs, metal oxides, and biomolecules for the electrochemical detection of H_2_O_2_ secreted from cancerous cells is still missing in the literature.

This review highlights the recent development in the application of carbon nanomaterials and metal nanoparticles for H_2_O_2_ detection. A subdivision of the sensors has been made depending on the nanomaterial used: (i) metal nanoparticles, (ii) graphene modified with metal or metal oxide nanoparticles to form “graphene nanocomposites,” (iii) enzyme-loaded graphene-like 2D nanomaterials, and (iv) carbon nanotubes modified with metal or metal oxide nanoparticles. Finally, the current potential and challenges of using carbon nanomaterials for H_2_O_2_ detection are outlined. Relative electrochemical properties such as limit of detection (LOD), sensitivity, and stability are reported for each sensor, and a critical comparison between the results has been carried out by summarizing the strengths and weaknesses of the various sensors found in the review.

## 2. Classical Methods for H_2_O_2_ Detection

### 2.1. *Electrochemical* *Systems*

**Figure 1:**
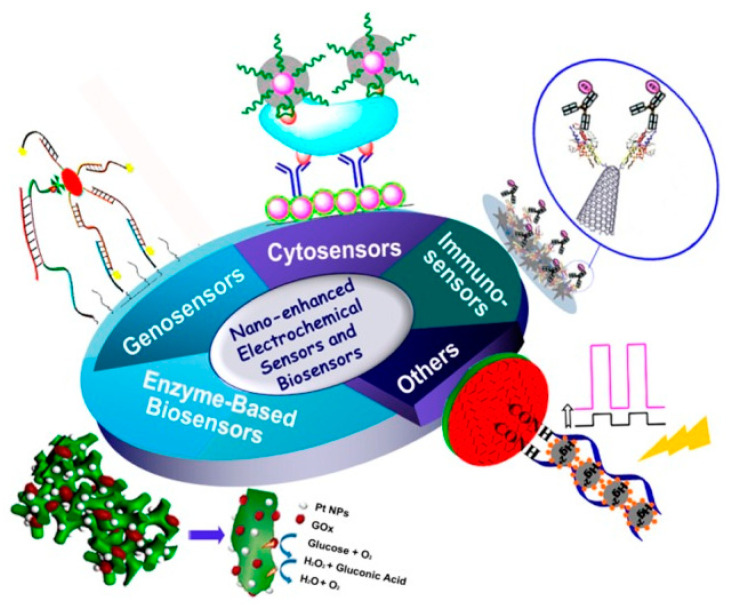
Distinct strategies for the electrochemical detection of H_2_O_2_ including Cyto-biosensors, Immuno-biosensors, Enzymatic and non-enzymatic biosensors. Figure reproduced with permission from [46]. Copyright 2014. American Chemical Society (Washington, DC, USA).

Electrobiochemical biosensors are built on the principle that they have a biorecognition component providing an electroactive constituent after reacting with an analyte that is transformed into an electrical signal that is measured using a physiochemical transducer as shown in Figure 1. The electrochemical transducers help in the detection and monitoring of the changes in the electrical current and potential. The commonly used easily detected biorecognition elements are enzymes. Antibodies, complete cells, and DNA can also be conventionally used for the construction of electrochemical biosensors as biorecognizable elements [1,42] by loading on a metal surface or carbon electrodes [43]. The most used electrochemical transducers are amperometric, potentiometric, conductometric, and impedimetric. The advantage of using electrochemical biosensors in analytical techniques is that they can be effectively used to reduce the size of the device. Additional advantages are that these sensors are low cost, highly sensitive, stable, and reproducible; show a linear response; can detect turbid samples; and are efficient due to using low sample volumes and chemical consumption [5,6,44,45]. 

### 2.2. Potentiometric

Biosensors act on the principle of measuring the electric potential generated by an electrode in the absence of a substantial amount of current via a reference and functional electrode that has been adjusted to sense selective analytes, with membranes that cover the electrode surface. When a target analyte interacts with the membrane covering the electrode surface, a subsequent change in electric potential is detected and measured by the electrode [47]. Potentiometric biosensors work by measuring the electric potential at zero current, which helps in differentiating the reference and functional electrodes. Potentiometric sensors measure the generated electric potential of ion-selective electrodes during biological reactions with target-specific ions. In potentiometric biosensors, the enzyme is attached on the surface of the electrode by glutaraldehyde crosslinking or an adsorption process. The probe of pH meter is covered by the membrane, where biological reaction either produces or absorbs hydrogen ions. The alteration in hydrogen ions causes a change in pH, which is a measure of the concentration of the analyte [48]. For instance, two example of potentiometric biosensors depending upon the type of electrode used are as follows: (a) Potentiometric biosensors use a Nafion membrane/Pt electrode for H_2_O_2_ determination, with an additional advantage of a perm-selective barrier [49]. Ascorbate and redox-active species reduce the overall electrode response, which further potentiates coupling between the redox potential on the Pt electrode and Donnan potential and increases sensitivity in detecting H_2_O_2_. The present potentiometric biosensor has a sensitivity of 125.1 ± 5.9 mV/decade, linear range of 10–1000 µM, and LOD of 10 µM [49]. (b) Zheng et al. used an MnO_2_/CPE to detect H_2_O_2_ [50]. The biosensor shows sensitivity of 19.4–121 mV/decade, with a wide linear range of 0.3–363 µM and LOD of 0.12 µM. The analytical parameters of the MnO_2_ doped/CPE biosensor were superior to those of the up-to-date potentiometric biosensors, i.e., Nafion membrane/Pt electrode [49], because of the enhanced electrode surface area, linear range, and LOD, except for the sensitivity. The coupling between the redox potential on the Pt electrode and Donnan potential made the Nafion membrane/Pt electrode superior to the MnO_2_ doped/CPE in terms of sensitivity [50].

### 2.3. Amperometric Biosensor

Zhao et al. developed an amperometric biosensor by immobilizing HRP on a silica sol–gel matrix on CPE for the determination of extracellular H_2_O_2_ excreted from breast cancer cells. The amperometric biosensor detected H_2_O_2_ via a sequence-specific peptide immobilized on the electrode surface and explicitly bound with horseradish peroxidase (HRP) in an auspicious orientation. The composed biosensors showed a linear range from 1.0 × 10^−7^ to 1.0 × 10^−4^ M, with a detection limit (LOD) of 3.0 × 10^−8^ M [51]. Zhao et al. showed a linear calibration of H_2_O_2_, i.e., 2 × 10^−5^ to 2.6 × 10^−3^ M, under optimum conditions [52]. In another strategy, a hydrogen peroxide biosensor was fabricated by coating a sol–gel–horseradish peroxidase LSPR layer onto a Nafion-methylene green modified electrode to develop a probe for H_2_O_2_ detection. The developed electrode exhibited sensitivity of 13.5 μA mM^−1^, with a detection limit of 1.0 × 10^−7^ M and response to 95% of the steady-state current in <20 s [53]. To detect H_2_O_2_, Tripathi et al. developed a novel biosensor by entrapping HRP in a new Ormosil composite doped with ferrocene monocarboxylic acid–bovine serum albumin conjugate and multiwalled carbon nanotubes (MWNTs). The developed biosensor showed a linear range of 0.02–4.0 mM, with a LOD of 5.0 μM (*S*/*N* = 3) [54]. In another study, a titania sol–gel matrix entrapping hemoglobin (Hb) was used as a peroxidase mimetic to sense H_2_O_2_ with a linear range from 5.0 × 10^−7^ to 5.4 × 10^−5^ M and a detection limit of 1.2 × 10^−7^ M [55]. Povedano et al. used His-Tag–Zinc finger commercial (His–Tag–ZFP) protein. The His–Tag–ZFP prefers to bind with RNA hybrids over ssRNAs, ssDNAs, and dsDNAs. These were further conjugated with streptavidin–HRP (Strep–HRP) in order to detect H_2_O_2_ with a LOD of 0.91 nM [56]. Reduced graphene oxide wrapped ZnMn_2_O_4_ microspheres (ZnMn_2_O_4_@rGO)-modified glassy carbon electrode (GCE) (ZnMn_2_O_4_@rGO/GCE) was used to make amperometric biosensors to detect H_2_O_2_. The resultant electrode showed a linear detection with a wide concentration range of 0.03–6000 μM and was used to detect H_2_O_2_ released from human breast carcinoma cells (MCF-7) as low as 0.012 μM [57]. Dong et al. reported a high-performance sensor using high-index facets of Au–Pd nanocubes loaded on large surface reduced grapheme oxide (rGO), and GCEs were modified by physical adsorption of both nanocomposites. The synthesized biosensor with three-dimensional nanocomposites possessed high sensitivity toward H_2_O_2_, with a minimum LOD of 4 nM, wide linear range from 0.005 μM to 3.5 mM, and swift response time [58]. Later, Jia and his coworker developed a nonenzymatic biosensor composed of poly(diallyldimethylammonium chloride) (PDDA)-capped rGO nanosheets loaded with a trimetallic AuPtAg nanoalloy. This biosensor detects H_2_O_2_ released from carcinoma cells with a LOD of 1.2 nM and a wide linear range from 0.05 μM to 5.5 mM [59]. In another study, hierarchical Mo_2_C@MoS_2_ consisting of interlayer-expanded MoS_2_ nanosheets wrapped on Mo_2_C nanorods was built as a highly sensitive, bifunctional electrochemical biosensor to detect H_2_O_2_ produced by cancerous cells, with sensitivity of 1080 μA mM^−1^ cm^−2^ and LOD of 0.2 μM [60]. Thiruppatthi et al. reported a simple stimulus responding aminophenol, pre-anodized screen-printed carbon electrode (SPCE*/AP) that could detect NADH and H_2_O_2_. The electrode was built by adsorbing aminophenol on the surface of the electrode, prepared from aminophenylboronic acid via boronic acid deprotection with H_2_O_2_. The resulting biosensor displayed linear ranges from 50 to 500 µM and from 200 µM to 2 mM, with detection limits (*S*/*N* = 3) of 4.2 and 28.9 µM for NADH and H_2_O_2_, respectively [61]. Maji et al. used cetyltrimethylammonium bromide-loaded gold nanorods (AuNRs) immobilized on a GC electrode to construct an amperometric biosensor (AuNRs/GC for the electrocatalytic detection of H_2_O_2_ under localized surface Plasmon resonance (LSPR) excitation (808 nm, 2 W cm^−2^). This biosensor showed an exaggerated improvement in its biosensing properties (~2–4-fold), with a wide linear range from 5.0 μM to 5.0 mM, LOD of 1.8 μM, and sensitivity of 1.6 μA mM^−1^ cm^−2^ [62]. In another study, self-supported MoS_2_ nanosheet arrays were built, and they showed highly potent electrocatalytic performance, with a LOD of 1.0 μM (*S*/*N* = 3) and high sensitivity of 5.3 mA mM^−1^ cm^−2^. This biosensor with self-supported MoS_2_ nanosheet arrays successfully detected trace amounts of H_2_O_2_ released from live A549 cancer cells [63]. 

### 2.4. Calorimetric Biosensors 

A new area of nanotechnology and its integration with biosensors has introduced the concept of calorimetric biosensors for cancerous cell diagnosis and detection. Li et al. used a microfluidic paper-based analytical device (μ-PAD) for the synchronous sensitive and visual detection of H_2_O_2_ released from cancer cells. μ-PAD construction was done using a layer-by-layer modification of concanavalin A, graphene quantum dots (GQDs)-labeled flower-like Au@Pd alloy nanoparticles (NPs) probe, and vertical alignment of cancerous cells on the surface of ZnO [64]. In the study by Zhang et al. porous platinum NPs on graphene oxide (Pt-NPs/GO) were used in building a calorimetric biosensor. The resultant nanocomposite functioned as a peroxidase mimetic, which could catalyze peroxidase substrate reaction in the presence of H_2_O_2_. Building on this phenomenon, Pt-NPs/GO acts as a signal transducer in developing a colorimetric assay for cancerous cell detection [65]. Additionally, porous, alloy-structured PtPd nanorods (PtPd PNRs) were used as a peroxidase mimetic for H_2_O_2_ detection. The PtPd PNRs were found to have a detection limit of 8.6 nM and a linear range from 20 nM to 50 mM and were used as a signal transducer to develop an innovative detection method for studying the flux of H_2_O_2_ released from cells [66]. Folate and iron-substituted polyoxometalate [(FeOH_2_)_2_SiW_10_O_36_] provided a novel method for the detection of H_2_O_2_ with good sensitivity, with a linear range of 1.34 × 10^−7^ to 6.7 × 10^−5^ mol L^−1^, and low detection limit (1 × 10^−7^ mol L^−1^) and swift response toward H_2_O_2_. Ye et al. showed a new analysis method based on calorimetric analysis. The colorimetric biosensing strategy was based on iodide-responsive Cu–Au nanoparticles (Cu–Au NPs) combined with the iodide-catalyzed H_2_O_2_–3,3,5,5-tetramethylbenzidine (TMB) reaction system. The bimetallic Cu–Au NPs absorbed iodide, thus indirectly inducing the colorimetric signal variation of the H_2_O_2_–TMB system. The results demonstrated economically effective, simple, label-free visualization of H_2_O_2_ from cancerous cells with high selectivity and sensitivity. The resultant calorimetric biosensor operates with a linear range from 50 to 500 cells/mL and a LOD of 5 cells in 100 μL [67]. Calorimetric biosensors can be designed using single wall nano tube (SWNT), which is subsequently embedded within a collagen matrix. When there is an angiogenic stimulation of human umbilical vein endothelial cells (HUVECs), H_2_O_2_ molecules are released, which can be detected using this SWNT sensor. The constructed calorimetric biosensor shows calibration from 12.5 to 400 nM and can measure H_2_O_2_ at a nanomolar concentration in HUVEC from humans, with 1 s temporal and 300 nm spatial resolutions [68]. Other biosensors based on the principle of calorimetry include a biosensor with an electrode made of a 2D hybrid material (RGO–PMS@AuNPs). This biosensor displayed remarkable electrochemical performance and possessed high sensitivity and high selectivity in detecting H_2_O_2_ in 0.1 M phosphate-buffered saline as compared to enzymatic biosensors. The developed biosensor has an additional advantage over other sensors because it is nontoxic and can detect H_2_O_2_ without any intrusion by common interfering agents, with high sensitivity of 39.2 μA mM^−1^ cm^−2^, broad detection ranges from 0.5 μM to 50 mM, and a LOD of 60 nM. The sensor has high efficiency and can detect H_2_O_2_ in trace amounts, i.e., as low as nanomolar, secreted from living HeLa and HepG2 tumor cells [69].

### 2.5. Chemiluminescence Material for the Detection of H_2_O_2_

**Figure 2:**
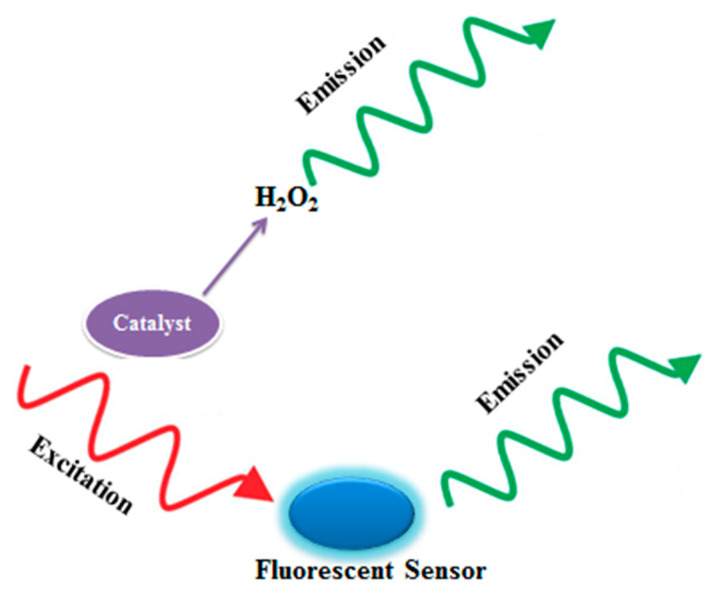
Mechanism of the chemiluminescent material for the detection of H_2_O_2_ released in cancer cells. Excitation and de-excitation of chemiluminescence materials can be seen during chemical reaction.

For the early diagnosis and detection of cancerous cells, it is important that molecules that indicate changes or biomarkers should be efficiently imaged and sensed. These parameters are important especially in studies that are evaluating the clinical mechanisms and designing effective chemotherapeutic agents [70,71]. The diagnostic and therapeutic methods for multiple detections are slow and need repetitive sampling, which results in low sensitivity and accuracy, because of heterogeneous sampling for separate detections [72]. The multiple fluorescence (FL) technique has promising results when used in in situ concurrent detection of multiple biomolecules. This technique has certain limitations such as weak compatibility with different biological systems, toxicity to living cells, and necessity for specialized synthesis and preparation [70]. Additionally, the FL signals generated using this technique faced interference, changes from background effects, and photobleaching while operating. Thus, it is highly desirable that in situ sequential detection of multiple biomolecules using within a complex biological sample is greatly desirable, the FL technique should be researched in cancer diagnostics without the current limitations [73]. The chemiluminescence (CL) technique is based on the principle that light is generated because of the energy released during a chemical reaction due to the de-excitation of the high energy moieties to the ground state or through energy transfer to luminophore molecules as shown in Figure 2 [74,75,76]. CL methods have gained popularity because these techniques are highly sensitive, are free of interference, phototoxicity, and photobleaching, and show no changes from background effects. The combination of CL methods with enzymes and analytes such as firefly luciferase (FFLuc) for 5′-triphosphate disodium salt (ATP) [77,78] and HRP [79,80] may result in a highly sensitive and competent method for H_2_O_2_ detection. In the recent advancement in the field of NPs, the multifunctional NPs with shell-like structures in their core have promising results in simultaneous diagnosis and treatment in living systems [81,82,83,84]. These multifunctional NPs were synthesized in the study by Ren et al. where dual functioning NPs were developed by combining HRPSiO_2_@FFLuc NPs with the enzyme-based core–shell structures, where the enzymes HRP and FFLuc were the main components of the core and shell of the NPs. They used the dual functioning NPs for the simultaneous in situ sequential detections and imaging of two biomolecules, namely, ATP and H_2_O_2_, in the same biological system. The surroundings of tumor cells or tissues are slightly acidic, and SiO_2_ is sensitive to an acidic environment, which causes the breakage of the SiO_2_ layer/component and exposes FFLuc and HRP (outside) and the SiO_2_ core (inside) to catalytic reactions. This results in the emission of two separate but simultaneous chemiluminescence signals for the sequential detection of ATP and H_2_O_2_, which avoids the signal interference between each other [73]. In another study by Lee et al., a novel contrasting agent was successfully synthesized, which was highly sensitive and specific and could image H_2_O_2_ in living systems [85]. The authors used peroxalate NPs to image H_2_O_2_ by inducing a chemiluminescent reaction using three components: H_2_O_2_, peroxalate esters, and fluorescent dyes. The peroxalate NPs were coated with peroxalate esters (hydrophobic polymer in its matrix). These NPs image H_2_O_2_ via a dual step process. Firstly, H_2_O_2_ diffusion occurs in the NPs, which then causes a reaction with the peroxalate ester groups and generates dioxetanedione, creating high energy inside the NPs [86,87], which subsequently then chemically excites the encapsulated fluorescent dyes, via a chemically initiated electron-exchange luminescence mechanism [88,89], leading to CL from the NPs and allows imaging of H_2_O_2_. Additionally, Lee et al. developed a method to synthesize peroxalate micelles, with a composition of amphiphilic peroxalate-based copolymers, rubrene (fluorescent dye), and a “stealth” polyethylene glycol (PEG) molecule to evade macrophage phagocytosis, which could successfully detect H_2_O_2_ through CL. These peroxalate-loaded micelles detected H_2_O_2_ within nanomolar concentrations (>50 nM) and were highly sensitive in detecting H_2_O_2_ in low physiological concentrations inside living systems [90].

Another study found that using peroxyoxalate chemiluminescent (POCL) NPs, H_2_O_2_ could be detected in trace amounts within living systems (in vivo) using optimized CL techniques in the near-infrared (NIR) wavelength. The detection of H_2_O_2_ using NIR is efficient in living systems because the penetration power of these NIR rays is higher because of the reduced photon-limiting interferences (scattering and absorption) happening within biological mediums [91,92,93]. CL using luminol was synthesized using o-benzyl alcohol-decorated block poly(carbonate)s copolymer, viz., PMPC–ONA, giving the resultant micelles a high H_2_O_2_ detection ability. In these micelles, luminol, fluorophore, and hemin were wrapped, forming an L/H/S@PMPC–ONA nanoprobe. These micelles work based on the principle that in the presence of H_2_O_2_ in the system, H_2_O_2_ diffuses within NPs, reacts with the hemin, and generates high energy reactive oxygen. The high energy reactive oxygen then chemically excites the luminol, activating the CL to expose nitrosobenzaldehyde recognition sites. This process destabilizes the micelles and releases the fluorescent indicator (fluorophore), which helps in imaging H_2_O_2_ [94]. Lee et al. additionally synthesized a nanoprobe using multiple molecule integration, i.e., dye/peroxalate NPs, which exhibited more enhanced and controlled CL, and hence displayed widespread applications in biomedical imaging of H_2_O_2_. This new enhanced nanoprobe was synthesized using nanoscopic coaggregation of a dye, which exhibited the aggregation-enhanced fluorescence phenomenon with a peroxalate, which had a high response to H_2_O_2_, which converted the energy generated from the chemical reaction to electronic excitation [95]. Additionally, Lee et al. successfully detected and imaged H_2_O_2_ via CL resonance energy transfer in the NIR wavelength using quantum dots functionalized with a luminol derivative [96]. Geng et al. devised a method to detect H_2_O_2_ via aggregation-induced emission fluorogen using 2,3-bis(4-(phenyl(4-(1,2,2-triphenylvinyl)-phenyl)amino)phenyl)-fumaronitrile (TPETPAFN), resulting in dye-encapsulated NPs [97]. A polyoxometalate (POM), vanadomolybdophosphoric heteropoly acid (H_5_PMo10V_2_O_40_, PMoV2), shows similar activity like peroxidases and functions by catalyzing the luminol/H_2_O_2_ reaction to generate CL. This phenomenon was shown in the study by Jia et al. where the study results showed an enzyme-free luminol/H_2_O_2_/PMoV_2_ CL system, which could be utilized for its high sensitivity in detecting H_2_O_2_. This enzyme-free luminol/H_2_O_2_/PMoV_2_ CL system exhibited good linear dependence with respect to H_2_O_2_ concentration within a wide range of up to 5 to 5000 nM (LOD) [98].

### 2.6. Titrimetry

The titrimetry technique can be used to analyze an unknown amount of H_2_O_2_ in a known sample concentration. The titrimetric technique uses iodometry, permanganate, and cerium (IV) in an acidic medium. In the study by Klassen et al., the concentration of H_2_O_2_ was assessed at 300 µM using the I_3_^−^ method after the calibration with permanganate. ε max measurement was made at 351 nm as 25,800 M^−1^ cm^−1^ using the calibration plot of the I_3_^−^ method titrated against potassium dichromate (KMnO_4_) [99]. In the study by Murty et al., the concentration of H_2_O_2_ was measured potentiometrically in an acidic medium using 8–11 M phosphoric acid [47]. Kieber and Helz synthesized a method for the detection of H_2_O_2_ by modifying the iodometric titration method using water matrices, where iodine was liberated as follows:

H_2_O_2_ + 2H^+^ + 2I^−^ → I_2_ + 2H_2_O

I_2_ + 2H_2_O + C_6_H_5_AsO = 2I^−^ + 2H^+^ + C_6_H_5_AsO(OH)_2_

The I_2_ produced was consumed by adding an excess of phenylarsine oxide. The end result was declared by titrating the remaining amount of phenylarsine oxide with iodine [100] when the intense blue color of the starch–iodine complex disappeared. The LOD was 0.02 µM. In another study, a two-step absorbance, microtiter plate method was developed by titrating an acidified H_2_O_2_ solution with standard cerium (IV) sulfate. In the second step, cerium (IV) sulfate was converted into cerium (III) sulfate, and potassium iodide was converted into iodine [101]. This process is commonly used and possesses additional advantages over the other methods because of its simplicity and low running costs, but its limitation is its inaccuracy at lower concentrations. Additionally, the other limitations of the method are that it consumes more time and requires skilled personnel to perform the calibration of the instrument.

### 2.7. Spectroscopy 

One of the most common, convenient, and extensively used methods for determining and measuring H_2_O_2_ is spectroscopy. This method is based on the principle that colored compounds are formed with respect to absorbance measurements comparative method of methyl blue and toluidine. A method comparing the reaction of methyl blue and toluidine blue with iodine solution was introduced for determination of H_2_O_2_ based on the following reaction:

H_2_O_2_ + 2KI + 2HCl → I_2_(aq) + 2KCl + 2H_2_O.

In the comparison, methyl blue when reacted with iodine gave a single-peak visible spectrum with a higher extinction coefficient (=49,100 M^−1^ cm^−1^) [102]. In another study by Matsubara et al., a method using a mixture of titanium IV and 2,4((5-bromopyridyl)azo)5-(*N*-propyl-*N*-sulfopropyl amino) phenol disodium for determining H_2_O_2_ [103] was demonstrated. Molar absorptivity was found to be 5.7104 M^−1^ cm^−1^ at 539 nm. In the study by Clapp et al., the measurement of H_2_O_2_ was done using an aqueous solution with titanium (IV) sulfate. This method yielded a yellow peroxotitanium species at a wavelength of 407 nm [104]. An in vitro method for the detection of H_2_O_2_ was developed using the 1,10-phenanthroline method. The advantages of this method are its short processing time, increased sensitivity, and high reproducibility [105]. In another study, catalytic decomposition of H_2_O_2_ was demonstrated by monomeric molybdenum (VI) by mixing hydroquinone, ammonium molybdate, and anilinium sulfate with varying H_2_O_2_ concentrations and determining the absorbance at 550 nm [106]. Zhang and Wong demonstrated a method for the estimation of the concentration of H_2_O_2_ in marine water at acidic pH of 4 in the presence of HRP at 592 nm using leuco crystal violet oxidation. The LOD for H_2_O_2_ was found to be 20 nM with ±1% accuracy [107]. In the study by Huang et al., a fast, reproducible, and reliable method for the detection and measurement of H_2_O_2_ was demonstrated. This method used 4AAP-DEA-βCD-hemin, and the LOD was 8.4 × 10^−5^, with a molar absorption coefficient of 1.65 × 10_4_ mol/L/cm [108]. Zhang et al. showed the determination of H_2_O_2_ in pulp bleaching effluents. The study shows that H_2_O_2_, in the presence of sulfuric acid solution, chemically reacted with vanadium pentoxide and formed a peroxovanadate complex that is reddish-brown [109]. 

### 2.8. Colorimetry

The method of determining H_2_O_2_ using iodide and starch was first developed in 1943 by Eisenberg. The H_2_O_2_ samples were treated with a titanium sulfate reagent, and the changes in color were quantified with the presence of H_2_O_2_. The chemical reaction of H_2_O_2_ with the titanium sulfate reagent is shown as follows:

Ti^+4^ + H_2_O_2_ + 2H_2_O = H_2_TiO_4_ + 4H^+^.

The formation of a yellow compound called pertitanic acid determined the H_2_O_2_ concentration within a range of 0.2–3.0 mg/100 mL [110]. Another study showed a more sensitive method using colorimetry. The study showed that the oxidation of iodide takes place in the presence of (NH_4_)_2_MoO_4_ (ammonium molybdate), which helps in determining the concentration of H_2_O_2_ even in micromolar quantities. The study determined the molar absorptivity of the starch–iodine complex (intense blue) at a value of 39.45 mmol^−1^ cm per liter at a wavelength of 570 nm [111]. The colorimetric method based on enzymes using plant extracts was developed by Fernando et al., where a sharp pink quinoneimine dye was formed. The pink dye formation took place when H_2_O_2_ reacted with phenol, 4-aminoantipyrine, and HRP in 0.4 M phosphate buffer with pH of 7.0 [112]. The assay results were considered optimum when the assay conditions were maintained at pH 7.0, temperature of 37 °C, 0.7 mM H_2_O_2_ concentration, and 1 U/mL enzyme concentration within 30 min. The optimum assay resulted in a limit of quantitation and LOD of 411 and 136 mM, respectively. Another simple method to detect the H_2_O_2_ released by cells within a tissue culture was based on the principle that phenol red oxidizes in the presence of H_2_O_2_. The study results showed a direct linear relationship between the concentration of H_2_O_2_ and absorbance, which had a range of 1 to 60 nmol/mL. The absorbance was measured at 520 nm [113]. Another fast and reliable method for determining H_2_O_2_ was developed using a colorimetry technique. In the method, 4-nitrophenylboronic acid was utilized for determining the concentration of H_2_O_2_ in an aqueous medium, where nitrophenylboronic acid reacted with H_2_O_2_ and produced 4-nitrophenol. The LOD was found to be ~1.0 μM [114]. Nitinaivinij et al. used the principle of colorimetry and demonstrated the determination of H_2_O_2_ in a very low concentration. The method utilized the technique of chromaticity analysis of silver nanoprisms (AgNPrs). The AgNPrs decomposed in the presence of H_2_O_2_, producing yellow color, and showed the H_2_O_2_ concentration at 1.57 mM with high accuracy and sensitivity [115]. The advantage of this method is that the determination of H_2_O_2_ can be carried out using a simple apparatus, but this method could give false-positive readings, and the results were not applicable to determine H_2_O_2_ within turbid samples.

### 2.9. Chromatography 

Chromatographic techniques are commonly used for separation. High-performance liquid chromatography (HPLC) is an analytical technique used for the detection and separation of different moieties. In the study by Takahashi, separation of H_2_O_2_ was achieved using an electrochemical detector and a cation-exchange resin gel column of sulfonated styrene-divinylbenzene copolymer. This method was found to have a linearity of 0.9984. The LOD was measured at 0.2 pmol [116]. In another method by Wada et al., H_2_O_2_ separation was achieved using an octadecylsilyl column, and the LOD was measured at 1.1 µM [117]. In another study, H_2_O_2_ was determined using gas chromatography in the presence of oxidized butyric acid, and its absorbance was found at a wavelength of 517 nm [118]. Another method in a study used a ligand exchange-type column for the separation of H_2_O_2_. The column was packed using a sulfonated polystyrene/divinylbenzene cation-exchange [119]. Steinberg et al. used the principle of reverse-phase chromatographic techniques in HPLC to determine H_2_O_2_. Iodovanillic acid was formed and was detected using UV absorption at 280 nm with a LOD of ~0.1 μM [120]. The advantages of chromatographic techniques in H_2_O_2_ determination are that these methods are relatively simple, have low operational costs, and use a wide range of stationary phases and columns. The limitations of this technique are its costly overall equipment, its long operational time, interferences, and the necessity for a specialized operator to run the machine. 

### 2.10. Fluorescence

**Figure 3:**
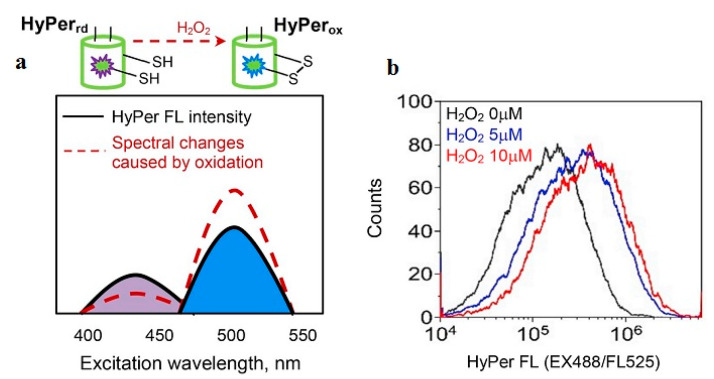
Analysis of HyPer fluorescence in K562 cells exposed to extracellular H_2_O_2_. (**a**) Scheme demonstrating the changes in the excitation spectrum of HyPer upon oxidation. (**b**) Flow cytometry histograms of K562 cells measured after two-minute exposure to different concentrations of H_2_O_2_. Reproduced with permission from [127]. Copyright 2019, Science Direct (Amsterdam, The Netherlands).

Another common method to detect H_2_O_2_ that has wide applications is based on the principle of fluorescent signal detection. In fluorescence sensors, the excitation of electrons is achieved from an external photon source, in contrast to CL, where light is generated via a chemical reaction [121]. Many fluorescent probes have been constructed using different materials. The probes include naphthofluorescein disulfonate [122], homovanillic acid [123], peroxyfluor-1 [124], peroxyresorufin-1 [124], single-walled carbon nanotubes [125], peroxyxanthone-1 [124], and phosphine-based fluorescent reagents [126]. In one study, the fluorescent biosensors helped in the detection of intracellular H_2_O_2_ in mice peritoneal macrophages [122]. In the study by Miller et al., three fluorescent probes that were detectable via confocal and two-photon spectroscopic methods from the peroxysensor family were successfully developed. Each fluorescent probe emitted at a different wavelength from the other, which allowed these probes to be used in various applications with respect to specific emitting wavelengths [124]. Recently, intracellular H_2_O_2_ concentration can be measured using HyPer, a genetically encoded H_2_O_2_ biosensor (Figure 3) [127]. HyPer is a chimeric protein [128] composed from the permuted yellow fluorescent protein (cpYFP) and H_2_O_2_-sensitive domain of the bacterial transcription factor OxyR, which is responsible for sensing H_2_O_2_ [129]. In the study by Belousov et al., an H_2_O_2_ sensor named HyPer was developed and studied. The HyPer sensor was successful in detecting an increase in H_2_O_2_ levels in HeLa cells during Apo2L/TRAIL protein-induced apoptosis (programmed cell death). This sensor also detected increased levels of H_2_O_2_ in cells taken from rat adrenal medulla (PC-12) that had been previously exposed to nerve growth factor [128]. The HyPer family includes five probes: HyPer [128], HyPer2 [130], HyPer3 [131], HyPer7 [132], and HyPerRed [133]. GEFIs of this family consist of a circularly permuted fluorescent protein (cpYFP for the numbered HyPers or cpmApple for HyPerRed) integrated via short peptide linkers into the bacterial transcription factor OxyR lacking a DNA-binding domain. Upon oxidation by H_2_O_2_, OxyR forms an intramolecular disulfide bond [134] that elicits conformational rearrangements. These rearrangements are then transmitted into the chromophore center of a fluorescent moiety of a GEFI, causing fluorescence alterations that can be subsequently detected. HyPer and its improved derivates, HyPer2 and HyPer3, contain cpYFP. cpYFP has two excitation peaks at 420 and 500 nm and a single emission peak at 516 nm. When the OxyR domain is oxidized by H_2_O_2_, the intensity of fluorescence excited at approximately 420 nm (*F*_420_) decreases, whereas the intensity of fluorescence excited at approximately 500 nm (*F*_500_) increases proportionally. A sensor readout is generated as a *F*_500_/*F*_420_ ratio [128]. 

Another study by Xu et al. showed a specific probe called Mito-H_2_O_2_, which is used to detect mitochondrial-associated H_2_O_2_ levels in HeLa cells. The study further showed that Mito-H_2_O_2_ was an effective, sensitive, and quick mitochondrial-targeted sensor [135]. Xiao et al. also developed another fluorescent probe called ER-H_2_O_2_ specifically for targeting the endoplasmic reticulum, which was equally effective, sensitive, and quick in the detection of H_2_O_2_. Xiao et al. induced apoptosis in both the organelles using L-buthionine sulfoximine, and both of these probes were tested for H_2_O_2_ specificity and selectivity [136]. Shen et al. developed a microfluidic method, which had droplets in combination with gold nanoclusters. This method was demonstrated to have high sensitivity for the detection of H_2_O_2_ secreted by a single cell. When a single cell was isolated using a microdroplet (with a volume of 4.2 nL), it can secrete H_2_O_2_, which causes florescent changes in HRP-gold nanoclusters with high specificity and high sensitivity of 200–400 attomole. The high throughput performance (~103 single-cell encapsulated microdroplets per minute) of the resultant microfluidic device makes it a powerful tool to investigate cell-to-cell heterogeneity in releasing H_2_O_2_ at the large scale, promising revelation of new knowledge to understand the biological role of H_2_O_2_ in tumor cells [137]. Moreover, Wang et al. fabricated a Ce6@Lum-AuNPs nanoprobe using green syntheses methods. They successfully loaded luminol-gold NPs with the fluorescent receptor Chlorin e6 (Ce6). The resultant fluorescent Ce6@Lum-AuNPs proved successful towards fluorescent bioimaging of cancerous cells [138]. 

## 3. Recent Advances

### 3.1. Current Approaches in the Construction of Biosensors

Over the past 200 years, the use of enzymes was common because of specific substrate sensitivity. However, enzymes are highly unstable and sensitive and are prone to denaturation caused by environmental changes such as pH and temperature. Therefore, recent studies have focused on using an artificial pseudo-catalyst instead of enzymes to overcome the drawbacks [139,140,141]. Denaturation of enzymes is common in enzyme groups such as peroxidases, catalases, monoamine oxidase, choline oxidase, uricase, and ascorbate oxidase. Peroxidases, also known as heme proteins, constitute the prosthetic group, i.e., ferriprotoporphyrin, and are usually found to have a molecular weight of 30 to 150 kDa [142,143]. Peroxidases are oxidoreductases and are produced by many animals, plants, and microorganisms. Peroxidases reduce H_2_O_2_ and help in the oxidation of aromatic amines, phenols, and organic and inorganic substrates [144] and are extensively utilized in biochemistry, enzyme immunoassays, wastewater treatment plants containing phenol compounds, synthesis of aromatic compounds, and removing H_2_O_2_ from food materials [143]. The application of peroxidase enzymes is extensive, and they are commonly used in analytical techniques for the detection of glucose [145], cholesterol [146], uric acid [147], H_2_O_2_ [148], alcohols [149], and phenols [144]. Peroxidase enzymes are also used in the pharmaceutical industry for the construction of biosensors for the detection of different drugs in the body. As previously mentioned, enzymes are prone to degradation; hence, the latest research involves the replacement of enzymes with pseudo-catalysts, i.e., inorganic/organic. These materials are chosen because of their low cost, stability, and convenience [150,151,152].

### 3.2. Electrochemical Sensing of H_2_O_2_ via Metal Nanoparticles

Nanotechnology has advanced, and there are many types of NPs available nowadays. NPs can be classified based on the nanomaterial used to synthesize them. Commonly available nanomaterials include metal NPs [153,154], carbon nanomaterials [155,156], and metallic oxide nanostructures [157]. Nowadays, NPs are used in manufacturing H_2_O_2_ electrochemical sensors, exhibiting distinctive electrical and catalytic properties toward the reduction or oxidization of H_2_O_2_ and having a broad range of stability based on the nanomaterial used in them. However, until now, most of the studies reporting cost-effective H_2_O_2_ detecting electrochemical sensors have a detection limit of the sub-micromolar level [38,158]. To detect H_2_O_2_ in cellular matrices, the electrobiochemical sensors should be sensitive enough to sense H_2_O_2_ concentration in nanomoles. Currently, those biosensors that are highly sensitive and have optimum H_2_O_2_ detection limits have been developed using HRP and metal nanoparticles [159,160], which decreases the long-term operational stability and increases the operational costs. In the study by Wang et al., real-time electrochemical detection of H_2_O_2_ via small MoS_2_ NPs in Raw 264.7 cancerous cells was performed. The resulting device had a detection limit lower than 2.5 nM and a wide linear range of up to five orders of magnitude [161]. In vivo monitoring of H_2_O_2_ secreted from living cells is essential in understanding cellular signaling pathways. The release of H_2_O_2_ from living cells is very low because the selective detection of H_2_O_2_ at a low level is challenging. To overcome this difficulty of detecting endogenous H_2_O_2_ from live cells, Dou et al. synthesized three hybrid metal nanoflower sensors for the detection and monitoring of H_2_O_2_ concealed from living MCF-7 cancerous cells. The three-hybrid metal Au–Pd–Pt nanoflower-decorated MoS_2_ nanosheet-modified sensors were developed using simple wet chemistry. The three-hybrid metal nanoflower sensors (Au–Pd–Pt/MoS_2_) show a synergetic increase in the electrocatalytic reduction of H_2_O_2_ with an ultrasmall detection limit as low as the sub-nanomolar level. Immobilization of aminin glycoproteins on the nanocomposite surface will result in an increase of its biocompatibility, which, in turn, enhances composite adherence to cells. This property of nanocomposites can be effectively used in future applications directed toward monitoring the secretion of H_2_O_2_ from living cells and cellular apparatus and may be utilized in developing highly efficient and sensitive cancer diagnostics sensors [162]. Sun et al. synthesized a dumbbell-shaped Pt_*x*_Pd_100–*x*_–Fe_3_O_4_ NP composite, which could effectively determine the secretion of H_2_O_2_ from Raw 264.7 cells with a detection limit of 5 nM [160]. Chang et al. developed a sensitive fluorescent assay to determine H_2_O_2_ with a wide linear range of 1 to 100 μM and detection limit of 0.8 μM. A fluorescent biosensor based on the inner filter effect (IFE) was manufactured using poly (vinyl pyrrolidone)-protected gold nanoparticles (PVP–AuNPs) and fluorescent BSA-protected gold nanoclusters (BSA–AuNCs). The BSA–AuNCs acted as an IFE fluorophore pair. The high extinction coefficient of PVP–AuNPs served as a dominant absorber and influenced the emission of the fluorophore in the BSA–AuNCs assay. The surface Plasmon resonance (SPR) of PVP–AuNPs was significantly enhanced with an increase in H_2_O_2_ concentration. The increased H_2_O_2_ then caused the significant induction of the fluorescent quenching effect of BSA–AuNCs [163]. Cui et al. showed a fast, simple, and reagent-free method for H_2_O_2_ detection. The study used luminol-reduced Au NPs for the determination of H_2_O_2_. The resulting biosensor had the electrochemiluminescence application in effectively determining the concentration of H_2_O_2_ within limits of 3 × 10^−7^–1.0 × 10^−3^ mol L^−1^ with a low detection limit of 1.0 × 10^−7^ mol L^−1^ (*S*/*N* = 3) [164]. 

Liu et al. synthesized porphyrin functionalized ceria (Por-Ceria) uniform nanoparticles as a calorimetric probe for H_2_O_2_ detection [165]. A nickel phosphide nanosheet array on a titanium mesh (Ni_2_P NA/TM) possesses superior analytical performance with a rapid retort time of <5 s. Manufactured biosensors showed high selectivity and stability, with a wide linear range of 0.001–20 mM, ultrasmall LOD of 0.2 μM (*S*/*N* = 3), and high sensitivity of 690.7 μA mM^−1^ cm^−2^ [166]. Small (10–30 nm) platinum nanoparticles (Pt-NPs) were prepared via protein-directed one-pot reduction. The resultant BSA/Pt-NPs composite shows colorimetric determination of H_2_O_2_ with a linear range from 50 μM to 3.0 mM, LOD of 7.9 μM, and visually detected lowest concentration of 200 μM [167].

Ultrathin silver nanosheets that can detect H_2_O_2_ with a LOD of 0.17 µM, linear range of 5–6000 μM, and fast response time <2 s were synthesized by Ma et al. The synthesized biosensors showed real-time determination of H_2_O_2_ released from living HeLa and SH-SY5Y cells, with high sensitivity of 320.3 µA mM^−1^ cm^−2^ [168].

The synergistic combination of p-type semiconductive channels of layered double hydroxides (LDHs) exhibited multifunctional properties, a distinctive morphology, and abundant surface active sites. The Fe_3_O_4_@CuAl NSs modified electrode exhibited excellent electrocatalytic activity toward H_2_O_2_ reduction. The projected biosensor revealed prominent electrochemical sensing of H_2_O_2_ with an extensive linear range of eight orders of magnitude and a low detection limit of 1 nM (*S*/*N* = 3) [169]. Copper(I) phosphide nanowires on 3D porous copper foam (Cu_3_P NWs/CF) were fabricated via electrochemical anodized Cu(OH)_2_ NWs to manufacture noble metal-free electrocatalysts. The Cu_3_P NWs/CF-based sensor exhibited first-rate electrocatalytic reduction of H_2_O_2_ with a detection limit as low as that achieved by noble metal-free electrocatalysts, i.e., 2 nM. The developed sensor assured sensitive and consistent determination of H_2_O_2_ excretion from living tumorigenic cells [170]. Xiong et al. developed a nickel phosphide nanosheet array on a titanium mesh (Ni_2_P NA/TM) using an economical and effective metal toward electrocatalytic H_2_O_2_ reduction. Ni_2_P NA/TM, being a nonenzymatic H_2_O_2_ sensor, presented superior analytical performance, with a swift response time <5 s and wide linear range of 0.001–20 mM. The resultant electrode exhibited high sensitivity of 690.7 μA mM^−1^ cm^−2^ and ultrasmall detection limit of 0.2 μM (*S*/*N* = 3) [166]. A Prussian blue nanocube-decorated molybdenum disulfide (MoS_2_-PBNCs) nanocomposite was designed for the electrochemical sensing of H_2_O_2_. Interestingly, a sensor for label-free sensing of carcinoembryonic antigen (CEA) can be constructed by using MoS_2_-PBNCs nanocomposites. The electrochemical response of the MoS_2_-based immunosensor was linear, with a CEA concentration range from 0.005 to 10 ng mL^−1^ and minimum recognition limit of 0.54 pg mL^−1^ [171].

### 3.3. H_2_O_2_ Detection Using Enzymatic Biosensors

Various analytical techniques, i.e., chemiluminescence [95], fluorescence [172], and electrochemistry [80,160], have been employed for the analysis of H_2_O_2_ at the cellular level. Among them, electrochemical sensors are an area of high interest and provide fast, economically effective, and real-time determination via a simple mechanism with ultrahigh sensitivity and selectivity. Electrochemical detection is considered a powerful tool for the determination of other electroactive metabolites such as glucose [173], dopamine [174], and O_2_ [175] secreted from live cells. The high selectivity and sensitivity of enzymes made them valuable for the electrochemical biosensing of H_2_O_2_. Horseradish peroxidase (HRP) enzymes draw considerable attention for the construction of electrochemical biosensors because of their efficient catalysis of H_2_O_2_ [176,177]. Wang et al. reported a highly sensitive sequence-selective DNA sensor composed of an HRP-labeled probe. The proposed biosensor successfully detected the K-ras gene, which is associated with colorectal cancer. Thiol (–SH) modified capture probe adsorbed chemically on the gold electrode via self-assembly and exhibiting a detection limit of 5.85 × 10^−12^ mol L^−1^, hybridization of nucleic acid (target DNA:K-ras gene), and a HRP labeled oligonucleotide detection probe can be achieved using the sandwich way method. Wang et al. developed an extremely sensitive sequence-selective DNA sensor on an HRP-labeled probe to detect the specific K-ras gene which is associated with colorectal cancer. At first, the capture probe modified with –SH was chemically adsorbed on the gold electrode by self assembly. Then, a complementary nucleic acid (target DNA:K-ras gene) was hybridized with an HRP labeled oligonucleotide detection probe in a sandwich way with a detection limit of 5.85 × 10^−12^ mol L^−1^ [178]. Bruno et al. developed horseradish peroxidase conjugated gold nano biosensors for detection of H_2_O_2_ released by prostate cancerous cells. The proposed biosensor can detect hydrogen peroxide (H_2_O_2_) in a wide linear range from 2 to 100 μM with a low detection limit of 0.01 μM [179]. A Cyt c loaded nanostructured TiO_2_ film was successfully prepared by Luo, which exhibits natural enzymatic activity toward H_2_O_2_, redox formal potential (E^0′^) of 108.0 ± 1.9 mV versus Ag|AgCl, and an heterogeneous electron transfer rate constant (ks) of 13.8 ± 2.1 s^−1^ [157]. To stabilize the enzyme model, Zhou et al. used an enzyme cytochrome c (Cyt c), to facilitate the transfer of electrons between the redox enzyme and electrode. Cyt c was immobilized stably into the molecular hydrogel to maintain its innate bioactivity toward H_2_O_2_. The use of Cyt c is a consistent methodology to regulate H_2_O_2_ at an optimized potential with high selectivity over other ROS, oxygen, metal ions, and ascorbic acid. The in vivo sensing of H_2_O_2_ from living cells, a small molecular hydrogel provides long-lasting stability and good reproducibility [180].

### 3.4. Carbon-Based Material for H_2_O_2_ Sensing

**Figure 4:**
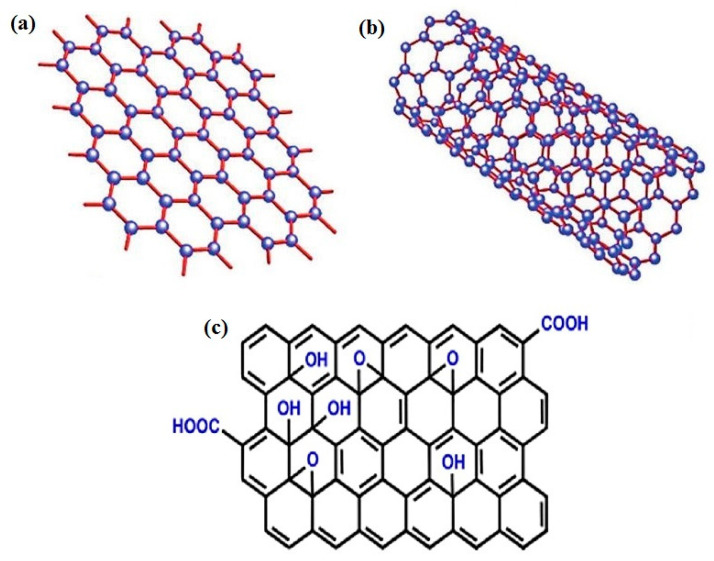
Different types of carbon-based materials, i.e., (**a**) Graphene, (**b**) Carbon nanotube, and (**c**) reduced grapheme oxide, used in electrochemical sensing of H_2_O_2_.

The success of graphene has boosted great research in the synthesis and characterization of graphene-like 2D materials, single and few-atom-thick layers of van der Waals materials, which show fascinating and useful properties. The single atom layer of C is the most transparent, strongest, and thinnest material and exhibits electrical conductance much better than Cu, with the ability to endure a current density that is six orders of magnitude [36,181,182]. The structure of some of the carbon-based material is shown in Figure 4. Recently, graphene has attracted great interest in the development of biosensors, i.e., optical and electrochemical, with improved performance owing to its integration with different nanomaterials (metals and metal oxides) and quantum dots [183,184,185]. Researchers have developed great interest in the emerging class of carbon-based 2D materials (graphene) because of their distinctive properties with applications in sensing and biosensing, electronics, catalysis, composites, and coatings. The excellent optical and electrical properties of carbon-based 2D materials made their use emergent in sensing and biosensing and showed real-time application in the field of biochemistry and nanomedicines [186,187]. Graphene-like 2D layered nanomaterials boron nitride (BN), transition metal dichalcogenides, graphite–carbon nitride (gC_3_N_4_), graphenes, and transition metal oxides have been investigated broadly [188,189]. Boron nitride nanosheets contain alternate nitrogen and boron atoms in a honeycomb lattice structure with extensive band gap, and BN is an insulator [190]. Instead of various transduction techniques, electrochemical methods are well known for analytical biomarker detection via graphene 2D-based sensors [191].

#### 3.4.1. Graphene-Based Metal-Free Electrocatalysts

The application of carbon materials in analytical and industrial electrochemistry is well known owing to their low cost and electrocatalytic potential in a number of redox reactions [192]. Recently, groups of researchers showed that surface functionalization of graphene materials results in diverse behavior, which made them benevolent in sensing in contrast to intrinsic graphene. Zhou et al. showed the chemical reduction of graphene oxide into chemically reduced graphene oxide (CR-GO) via hydrazine, and the resultant GCE constructed from the obtained CR-GO showed excellent sensing capability for H_2_O_2_ detection. The synthesized electrochemical biosensor exhibited a lower detection limit of 0.05 μM and wide linear range from 0.05 to 1500 μM, which precedes the use of functionalized carbon materials in electrochemical sensing [155]. 

In another work, Takahashi et al. reported rGO modified GCE via electrodeposition. The electrochemical studies showed an enhancement in the sensing performance of the rGO modified electrode that was considerably better than the original electrode for hydrogen peroxide detection. Some studies showed a high electron density on the defective sites (edges) of modified graphene oxide, which made it a potential candidate for the electrocatalytic reduction of H_2_O_2_ [193]. The synthesis of novel quality graphene is important in exploiting graphene application for electrochemical sensing. Chemical and physical (thermal method) reduction of GO (hydrophilic GO to hydrophobic graphene) is the most effective method to manufacture graphene on a large scale. During chemical and physical reduction, exfoliated graphene becomes disorderly aggregated, which results in the decrease in their disperse behavior in water and limits their practical applications [194]. Later on, some researchers fixed this problem using various dispersants, i.e., sodium dodecyl sulfate, cetyltrimethyl ammonium bromide (CTAB), and DNA. These dispersants enhanced the disperse behavior and stability of graphene in an aqueous environment. Lv et al. simply introduced DNA molecules on the graphene surface using the self-assembly method and formed graphene–DNA hybrids (GN/DNA). DNA–graphene was found to show a physical interaction, i.e., π–π stacking via aromatic rings of graphene and *N*-containing functional moiety in DNA, which results in a strong interaction between graphene and DNA. Stacking DNA on the graphene surface not only enhanced graphene dispersion in aqueous media but also imparted an electron-rich character to graphene by forming a GN/DNA composite. Comparative studies showed that the GN/DNA modified electrode exhibited higher sensitivity, wide detection range, and swift response time in contrast to the GN-modified electrode for the electrochemical sensing of H_2_O_2_ [195]. Woo et al. fabricated a multiwalled carbon nanotube–graphene composite (MWCNT–graphene) via a direct in situ chemical reduction of graphene oxide and pre-treated MWCNT mixture. The prepared component showed a uniform network of ultrathin graphene sheets stuck between nanotube bundles. Structural analysis showed that the morphology of graphene present between nanotube bundles was comparatively higher than pure graphene, which showed wrinkled and aggregated morphology. The electrochemical sensor constructed from the resultant MWCNT–graphene exhibited a wide detection range from 20 μM to 2.1 mM and low detection limit of 9.4 μM. Synergic increase in the electrochemical performance of the MWCNT–graphene composite is attributed to high electrical conductivity of MWCNTs [196]. Recently, metal-free electrocatalysts, heteroatom-doped graphene, play a crucial role in H_2_O_2_ detection. The electronic properties of graphene can be altered drastically by doping graphene with N, S, and B, which play a crucial role in operating the electronic properties. Wang and coworkers used the nitrogen plasma treatment strategy to produce N-doped graphene from reduced graphene oxide as a starting material. Spectral studies of N-graphene showed that the nitrogen atom was substituted into graphene sheets with three different nitrogens, including graphitic N, pyridinic N, and pyrrolic N. The concentration of nitrogen in graphene sheets was optimized by monitoring the plasma exposure time, and the resultant N-doped graphene showed improved electrocatalytic performance as compared to pristine graphene in electrochemical sensing [197].

Wu et al. reported the synthesis of N-doped graphene using hydrazine as a nitrogen source, with a 4.5% N/C atomic ratio, and reducing agent. Structural studies of N-doped graphene were made via XPS measurements. Structural analyses showed 28% pyridinic N, 49% pyrrolic N, 19% graphitic N, and 4% oxidized N [198]. Increased sensitivity, a wide linear range, and a low detection limit were achieved using N-doped graphene as compared to pristine graphene. In addition to N, Yeh et al. successfully synthesized boron-doped graphene nanosheets (BGNs) using B_2_O_3_ and graphene nanosheets through an atmospheric-pressure carbothermal reaction. Boron doping on the graphene surface created defects in nearby sites and uneven charge separation, which, in turn, facilitated the charge transfer to neighbor atoms. The resultant BGN-doped graphene showed a wide linearity range from 1.0 to 20.0 mM, detection limit of 3.8 μM, and much higher sensitivity (266.7 μA mM^−1^ cm^−2^) compared with undoped GNs [199]. Recently, the electrochemical performance of the detection of H_2_O_2_ was further improved using co-doped graphene with two elements. Yang et al. synthesized N and B co-doped graphene (NB-G) using a microwave-activated chemical–thermal treatment strategy. In this strategy, they first developed N-graphene using GO and cyanamide as a precursor, followed by microwave treatment. The boron atom was doped on N-modified graphene via the pyrolysis of the N-G and B_2_O_3_ mixture at 900 °C for 0.5 h in an Ar atmosphere to obtain BN-G [200]. Electrochemical studies of NB-G were made using ferric/ferrous coupling of K_3_[Fe(CN)_6_]/K_4_[Fe(CN)_6_]. The prepared electrode exhibited outstanding electrocatalytic reduction of H_2_O_2_ and a rapid response time, with a linear range from 0.5 μM to 5 mM and detection limit as low as 0.05 μM. The excellent electrochemical performance of the NB-G electrode is attributed to the novel structural network, with high charge transfer and large surface area, and the synergistic effect between the two heteroatoms of B and N [200]. Table 1 shows electrochemical performance of non-enzymatic metal free H_2_O_2_ sensors based on graphene.

#### 3.4.2. Carbon Composite with Enzymes for H_2_O_2_ Detection

Noble metals, nonnoble metal oxide, and sulfide-modified graphene composites are used to immobilize HRP for the construction of enzymatic H_2_O_2_ biosensors [212,213]. Song at al. [214] reported MoS_2_–graphene (MoS_2_-Gr)-based biocompatible biosensors for the ultrasensitive detection of H_2_O_2_. MoS_2_-Gr nanosheets were prepared using GO and NaMoO_4_ as precursors using the solvothermal method, and a change in solution color from reddish-brown (GO) to black confirmed the dispersion of dark flower-like MoS_2_ nanoparticles on the Gr surface. Structural analyses were made using XRD results, which confirmed the formation of MoS_2_-Gr composites. Electrostatic interaction arose between negatively charged MoS_2_-Gr nanosheets and positively charged HRP and resulted in the formation of the HRP-MoS_2_-Gr composite. The appearance of the peak in the UV–Vis spectra at 402 nm confirmed the immobilization of HRP on MoS_2_-Gr, whereas no peak was noticed in case of MoS_2_-Gr nanosheets. The HRP-MoS_2_-Gr fabricated biosensor showed excellent stability and enhanced electrocatalytic performance for H_2_O_2_ detection. The resultant biosensors exhibited a low detection limit of 0.049 μM and broad linear range from 0.2 μM to 1.103 mM. 

**Figure 5:**
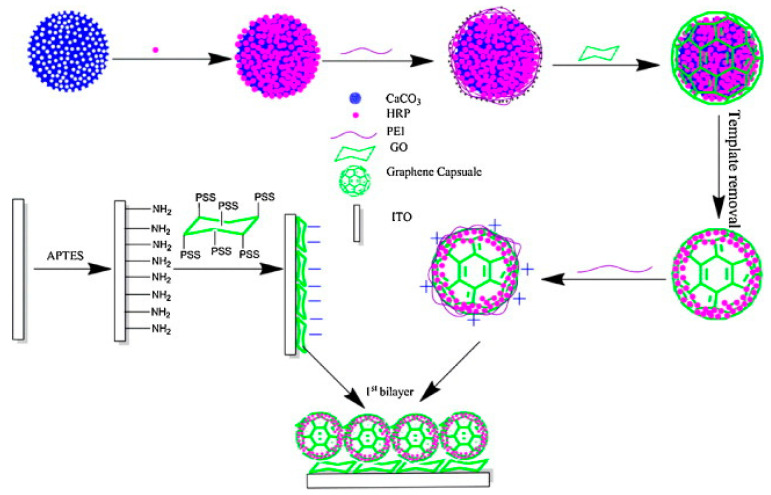
Mechanism of the synthesis of the graphene enzyme composite for the electrochemical sensing of H_2_O_2_. Reproduced with permission from [216]. Copyright 2015, Science Direct.

Later, Yu et al. immobilized horseradish peroxidase (HRP) on Au-decorated graphene oxide. The fabricated biosensors showed a fast response with remarkable performance, such as low detection limit (7.5 × 10^−9^ M) and real-time measurement of cellular H_2_O_2_ in living cells [27]. Liu et al. used horseradish peroxidase (HRP) immobilized on 3D porous graphene (PGN) to develop a real-time biosensor for the detection of H_2_O_2_ from living cells. Nanoporous graphene plays a significant role in the excess absorption of HRP, accelerates the diffusion rate, and shows excellent electrochemical performance toward H_2_O_2_ with a LOD of 0.0267 nM and wide linear range of seven orders of magnitude [215]. Enzymatic biosensors suffer from two major problems, namely, enzymatic loss and inactivation, which greatly affect biosensor performance. Fan and his coworker overcame this problem by encapsulating horseradish peroxidase on biomimetic graphene capsules (GRCAPS) using CaCO_3_ as a porous sacrificial template to mimic the existence form of bioenzymes in organisms as shown in Figure 5. As a result, the synthesized biosensor showed a low detection limit of 3.3 mmol L^−1^ and wide linear range of 0.01–12 mmol L^−1^ [216]. Wu et al. used another strategy to construct horseradish peroxidase–attapulgite nanohybrids on glassy carbon to fabricated biosensors. The prepared biosensor showed a rapid response, high sensitivity, and a low detection limit with a wide linear range for the detection of hydrogen peroxide released from RAW 264.7 macrophage cells [217]. Table 2 shows electrochemical performance of enzymatic H_2_O_2_ biosensor loaded on graphene.

#### 3.4.3. Graphene Composite with Metal Nanoparticles for H_2_O_2_ Detection

Dai et al. prepared heterogeneous Co_3_O_4_ dodecahedrons that contain carbon, and encapsulated Au nanoparticles (Au@C-Co_3_O_4_) were proposed via the pyrolysis of Au nanoparticle-encapsulated zeolitic imidazolate framework-67 (Au@ZIF-67). A remarkable increase in electrocatalytic performance with ultrahigh sensitivity of 7553 μA mM^−1^ cm^−2^ and with a detection limit of 19 nM was observed using the electrode fabricated from the porous Au@C-Co_3_O_4_ even with the 0.85% Au content in the composite. The synthesized biosensors were applicable for monitoring H_2_O_2_ concentration, which will be helpful in identifying cancerous cells [237]. A metal organic framework consisting of porphyrinand iron metal decorated on well-ordered mesoporous carbon (OMC) for hydrogen peroxide (H_2_O_2_) secreted from viable cells. Porphyrinic iron metal-organic framework (pFeMOF)-decorated ordered mesoporous carbon (OMC) was developed to detect hydrogen peroxide (H_2_O_2_) released from viable cells. Increased stability and electrical conduction were noticed with the introduction of OMC. Electrocatalytic reduction of H_2_O_2_ was observed at two different linear ranges, i.e., from 70.5 to 1830.5 μM and from 0.5 to 70.5 μM, with high sensitivity of 67.54 μA mM^−1^ at a low concentration and 22.29 μA mM^−1^ at a high concentration and with a detection limit (LOD) as low as 0.45 μM [238]. A nonenzymatic H_2_O_2_ electrochemical sensor was developed by immobilizing 2D ultrathin MnO_2_ nanosheets onto glassy carbon electrodes (GCE) with a Nafion film. The amperometric study showed an excellent increase in electrocatalytic reduction of H_2_O_2_ with an extreme low detection limit (5 nM), wide linear range (25 nM^−2^ μM and 10–454 μM), and high sensitivity of 3261 mA M^−1^ cm^−2^ via the immobilization of the MnO_2_ nanosheets. The constructed biosensors were efficaciously employed for real-time monitoring of H_2_O_2_ released from SP2/0 cells in trace amounts [239]. 

The functionalized hollow-structured nanospheres (HNSs) centered on Pd nanoparticles (NPs) adorned double shell-structured N-doped graphene quantum dots (N-GQDs)@N-doped carbon (NC) HNSs, with ultrafine Pd NPs and “nanozyme” N-GQDs as dual signal-amplifying nanoprobes, act as an exceedingly effective electrochemical sensor for the detection of H_2_O_2_ released from cancer cells. The hybrid HNS material-based synthesized electrochemical biosensors demonstrate excellent performance, which involves an ultrasmall detection limit as low as nanomolar and a rapid response time. The extra sensitivity, selectivity, and reproducibility of the synthesized biosensors make them valuable for real-time tracking of H_2_O_2_ released from different living cancer cells in a normal state and treated with chemotherapy and radiotherapy [26]. Heteroatom-doped graphene (N and B) exhibits multidimensional electron transport pathways, which make their use valuable in electrocatalytic sensing of H_2_O_2_ with excellent stability and response time. Table 3 and Table 4 show electrochemical performance of graphene-supported non-noble metal and noble metal nanoparticles.

The resultant N and B co-doped graphene (NB-G)-based electrochemical sensor showed a linear response from 0.5 μM to 5 mM with a LOD of 0.05 μM (*S*/*N* = 3). This increase in sensitivity with an ultralow detection limit to microlevel attributed to the NB-G constructive structure and special effects arose from the co-doping of N and boron in graphene [200]. CuFe_2_O_4_ nanoparticle-doped reduced graphene oxide based on a CPE was used as a voltammetric sensor for hydrogen peroxide (H_2_O_2_) sensing. The synthesized sensors showed a rapid amperometric response in less than 2 s and wide linear range of 2 to 200 μM with a low detection limit of 0.52 μM under optimum conditions (pH 5) [264]. Xi et al. synthesized N and S dual-doped graphene (NSG) co-doped carbocatalyst via one-pot syntheses. The NSG-modified electrode showed superior catalytic activity toward sensing, including a linear range up to 1.7 mM. ZnMn_2_O_4_-wrapped reduced graphene oxide microspheres (ZnMn_2_O_4_@rGO) act as an excellent electrocatalyst for H_2_O_2_ reduction. The ZnMn_2_O_4_@rGO-pasted glassy carbon electrode (ZnMn_2_O_4_@rGO/GCE) displayed a linear detection range of 0.03–6000 μM with a detection limit of 0.012 μM. The resultant biosensor showed promising results in physiology and diagnostics and was applicable in the determination of H_2_O_2_ secreted from human breast cancer cells (MCF-7) [57]. An AuNPs-NH_2_/Cu-MOF composite was prepared via ammoniation of Au NPs, anchored with a Cu-based metal organic framework (Cu-MOF). The synthesized AuNPs-NH_2_/Cu-MOF composite was further modified with a GCE to prepare an AuNPs-NH_2_/Cu-MOF/GCE electrode. The synthesized AuNPs-NH_2_/Cu-MOF/GCE composites possessed high sensitivity and selectivity, and they can be used as an electrochemical enzyme-free sensor for the quantitative detection of H_2_O_2_. Instead of quantitative H_2_O_2_ detection, the synthesized electrochemical sensor showed a wide linear response toward H_2_O_2_ concentrations ranging 5–850 μM with a LOD down to 1.2 μM [265]. Wang et al. improved the sensitivity of the electrode using hemin-capped biomineralized gold nanoparticles (Hem@AuNPs)-doped reduced graphene oxide (rGO), followed by coating with chitosan (CS). The resultant electrode from the prepared nanohybrids showed excellent electrocatalytic reduction of H_2_O_2_ with superior sensitivity, stability, and response time of few seconds. The most important feature of the synthesized electrode from the resultant nanohybrid is its lower detection limit of 9.3 nM and linear range of five orders of magnitude. Such characteristics enable this biosensor to detect H_2_O_2_ releasing from living Hela cells accurately and make this biosensor valuable for ultrasmall detection of H_2_O_2_ from living HeLa cells precisely [266]. Sun and his coworkers designed a novel nonenzymatic hydrogen peroxide sensor using intermetallic PtPb nanoplates (PtPb/G) supported on graphene with enhanced electrochemical performance for H_2_O_2_ detection in neutral solution and H_2_O_2_ released from the cells. The nanocomposite exhibited excellent electrocatalytic activity for the electrochemical reduction of H_2_O_2_ in half-cell test and with wide linear detection range of 2 nM to 2.5 mM and ultralow detection limit of 2 nM. An experiment further showed that the sensitivity of intermetallic PtPb nanoplates is 12.7 times higher than that of a commercial Pt/C electrode for the detection of H_2_O_2_ released from Raw 264.7 cells [267]. A graphene/Nafion/azure/I/Au nanoparticle composites modified glass carbon electrode (graphene/Nafion/AzI/AuNPs/GCE) was used for the construction of a nonenzymatic H_2_O_2_ sensor. The performance of the synthesized biosensors was recorded under optimum conditions, i.e., pH of 4.0 and potential of −0.2 V, upon the addition of H_2_O_2_. A stable current was obtained in less than 3 s, with a detection limit of 10 μM (*S*/*N* = 3) and a linear range of 30 μM to 5 mM [268]. Ju et al. reported a green and simple strategy for the in situ growth of surfactant-free Au nanoparticles (Au NPs) on nitrogen-doped graphene quantum dots (Au NPs–N-GQDs). The reported strategy showed the in situ formation of the Au NPs–N-GQDs hybrid by simple mixing of N-GQDs and HAuCl_4_·4H_2_O without any reductant and surfactant. The prepared nanocomposite (Au NPs–N-GQDs) exhibited a low detection limit of 0.12 μM and sensitivity of 186.22 μA/mM cm^2^ for the electrochemical detection of hydrogen peroxide (H_2_O_2_) [79]. Another research group developed a microelectrode with high sensitivity, a wide linear range, and good selectivity for the detection of H_2_O_2_ released from female cancer cells. The synthesized hierarchical nanohybrid microelectrode was composed of 3D porous graphene enfolded activated carbon fiber (ACF). This technique, i.e., green ionic liquid (IL), plays a crucial role in the simultaneous superficial and effective electrodeposition and electrochemical reduction of GO nanosheets on ACF to form a 3D porous ionic liquid functionalized electrochemically reduced GO (ERGO)-wrapped ACF (IL–ERGO/ACF) [269]. 

**Table 4:** Graphene supported noble metal nanoparticles for electrochemical detection of H_2_O_2_.

Graphene Based Material	Sensitivity μA mM^−1^ cm^−2^	Linear Range (μM)	Detection Limit (μM)	Ref.
Au-PEI/GO	460.0	0.5–1680	0.2	[270]
AgNPs-MWCNT-rGO	0.833	100–100,000	0.9	[271]
RGO-Au-PTBO	63.39	5.0–1077.1	0.2	[272]
Ag-MnOOH-GO	59.14	0.5–17,800	0.2	[273]
Au NPs@POM-G	58.87	5.0–18,000	1.54	[274]
AgNPs-TWEEN-GO	0.7459	20–23,100	8.7	[41]
GO-ATP-Pd	504.85	0.1–10,000	0.016	[275]
GN/Au-NPs	-	0.5–500	0.22	[276]
GN-Pt	0.01	2–710	0.5	[277]
Ag NWs-graphene	12.37	10.0–34,300	1.0	[278]
GR-AuNRs	389.2	30–5000	10	[279]
Au@C-Co_3_O_4_	7553	-	0.019	[237]
Au NPs-N-GQDs	186.22	0.25–13,327	0.12	[79]
AuNPs-NH2/Cu-MOF/GCE	1.71	5–850	1.2	[265]
GO/Au@Pt@Au	-	0.05–17,500	0.02	[280]
NG-hAuPd	5095.5	0.1–20	0.02	[281]
PDA-RGO/Ag NP	0.0111	0.5–8000	2.07	[282]
AgNPs/GN	-	100–100,000	0.5	[283]
Ag/SG	-	100–136,500	0.14	[284]
Pt/PG	341.14	1–1477	0.5	[285]
PDDA-RGO/MnO_2_/AuNPs	1132.8	5.0–500	0.6	[286]
AgNP/rGO	-	100–80,000	7.1	[287]
AgNPs-GO	-	10–20,000	0.5	[288]
RGO-AuNP	5.3	250–22,500	6.2	[289]
GNPs/SGS	27.7	20–15,000	0.2	[290]
AgNPs-CNT-rGO	-	10–10,000	1.0	[291]
PpyNFs/AgNPs-rGO	0.7367	100–5000	1.099	[292]
polystyrene@RGO-Pt	0.0675	0.5–8000	0.1	[41]
Graphene/Nafion/Azl/AuNPs	-	30–5000	10	[268]
Pt/GN	0.0204	2.5–6650	0.8	[41]
RGO-AuNPs (B)	9.5	25–41,500	5.0	[273]
PtAu/G-CNTs	313.4	2.0–8561	0.6	[293]
PtAuNPs-CTAB-GR	0.1654	0.005–4.8	0.0017	[294]
PtAu/RGO	4.105	0.015–8.73	0.008	[295]
Pt/graphene-CNT paper	1.41	0–25.0	0.01	[296]
pFeMOF/OMC	67.54	70.5–1830.5	0.45	[238]
Pd-PEI/GO	-	0.5–459	0.2	[297]
Pd-NPs/GN	0.019	0.001–2000	0.0002	[298]
PdNPGNs	2.75	0.1–1000	0.05	[299]
RGO-PMS@AuNPs	39.2	0.5–50,000	0.06	[69]
2Au1Ag-PDA/CFME	12966	0–55	0.12	[300]
TiO_2_NTs/r-GO/AgNPs	1152	15,500–50,000	2.2	[301]
PtPb/G	-	2–2.5	0.02	[267]
3DGA-AuNPs/cytc/GCE	351.57	-		[302]
PdPt NCs@SGN/GCE	-	1–300	0.3	[303]
AuNFs/Fe_3_O_4_@ZIF-8-MoS_2_	-	5–120	0.9	[304]

#### 3.4.4. Graphene-Loaded Biomolecules for Selective Detection of H_2_O_2_

Recently, graphene-based heme protein electrodes have gained wide attention for H_2_O_2_ detection. These graphene-based materials offer an appropriate microenvironment to maintain the redox bioactivity of proteins and make the transfer of electrons feasible between redox proteins (active centers) and the principal electrode [232]. A mixture of a strong acid and an oxidizing agent is used for the synthesis of graphene oxide from graphite [289]. GO serves as a precursor of graphene and as a sensing element. Several proteins, including cytochrome c, horseradish peroxidase (HRP), and myoglobin (Mb), were incubated. Zuo et al. [227] fabricated a heme proteins-modified GO electrode from GO suspension. Immobilization of protein on a GO sheet is associated with strong hydrophobic and electrostatic interactions between proteins and GO. The innate characteristics of the proteins remain unaltered in the presence of GO, which offers an appropriate microenvironment for the immobilization of protein with an intact structure. Studies revealed that the protein-based GO modified electrodes have an advantage over the featureless voltammograms because of the emittance of redox peaks from proteins on these electrodes, which stipulate an efficient electrical wiring of the redox centers of proteins to the surface of the electrode in the presence of GO. Importantly, the proteins retained their intrinsic peroxidase activity upon forming mixtures with GO and the catalytic properties provide a high sensing performance for H_2_O_2_ detection with low detection limit and wide detection range. Furthermore, Mani and coworkers improved the performance of Mb-based H_2_O_2_ biosensors using an RGO-MWCNT-Pt/Mb electrode [233]. The RGO-MWCNT-Pt composite was prepared using the wet chemical method, which provides good affinity and a large surface area for the accumulation of excess Mb. The Pt nanoparticles in the RGO-MWCNT-Pt/Mb composite showed excellent electrocatalytic activity and efficiently prohibited the accumulation and restacking of graphene sheets and CNTs. The resultant electrode (RGO-MWCNT-Pt/Mb) showed an excellent wide linear range from 10 pM to 0.19 nM with a detection limit of 6 pM and much higher sensitivity (1.99 μA pM^−1^ cm^−2^) compared to other biosensors. 

Additionally, HRP-fabricated H_2_O_2_ electrochemical biosensors were prepared using nano-graphene for the direct electron transfer from HRP to the substrates (electrode) [305]. These HRP-anchored graphene-based materials determine H_2_O_2_ with higher selectivity and sensitivity [224,306]. Zhang et al. reported immobilization of HRP and lysozyme enzymes on graphene oxide sheets in phosphate buffer solution by incubating GO with enzymes at 4 °C. The immobilized enzyme molecules were studied in situ using AFM, which clearly disclosed HRP molecules (bright spots) on the surface of GO. Strong hydrogen bonding and electrostatic interaction play a key role in loading enzymes (HRP and lysozyme) on graphene oxide, which was much higher than that on previously reported studies and was found to be the optimum solid substrate for the immobilization of the enzyme [307]. Moreover, Fan and coworkers applied the same method to generate graphene-poly (sodium 4-styrenesulfonate)/biomimetic graphene capsules (GS-PSS/GRCAPS) nanocomposites for direct electrochemical sensing of H_2_O_2_. Initially, porous CaCO_3_ was used as a support for HRP encapsulation in GRCAPS. Afterward, a GS-PSS/GRCAPS composite was synthesized via layer-by-layer electrostatic self-assembly, in which negatively charged GS-PSS electrostatically interact with positively charged PEI@GRCAPS [261]. GRCAPS was revealed to mimic the existing enzymes in living cells and provide a satisfactory microenvironment for HRP to realize direct electron transfer at the modified electrode. The resultant electrochemical biosensor exhibited long-term stability, low detection limit, extensive linear range, and an excellent anti-interference ability. A nonenzymatic and highly electrocatalytic H_2_O_2_ biosensor was proposed using a novel electrode composed of hemin-capped biomineralized gold nanoparticles (Hem@AuNPs), rGO, and chitosan (CS). The excellent rGO conductivity and outstanding electrocatalytic performance of Hem@AuNPs make them suitable for developing ultrasensitive biosensors for real-time determination of H_2_O_2_. Taking advantages of the peroxidase-like activities of nanohybrids, the resultant electrode demonstrated a highly selective and outstanding electrochemical performance toward H_2_O_2_ with fast response, improved sensitivity, and stability. More significantly, the lower determination limit of 9.3 nM and wider linear ranges of five orders of magnitude enable this biosensor to accurately detect H_2_O_2_ released from living HeLa cells [266]. Jiao et al. reported nonenzymatic biosensors for dynamic, most significant ROS. Intracellular nonenzymatic monitoring of H_2_O_2_ was achieved via loading of AuPtAg nanoalloy on rGO capped with poly (diallyldimethylammonium chloride). The constructed biosensor showed rapid and precise measurement of H_2_O_2_ released from cancerous cells. The precise and accurate detection of H_2_O_2_ is due to the remarkable rGO and PDDA conductivity with outstanding synergistic electrocatalytic performance of ternary alloys. The remarkable electrochemical performance of the resultant biosensor, with a low detection limit (1.2 nM) and wide linear range (from 0.05 μM to 5.5 mM), is due to the peroxidase-like activity of the AuPtAg nanoalloy [59]. In another study, a cytochrome c (Cyt c)-immobilized Au nanoparticle-loaded 3D graphene aerogel (3DGA) was synthesized for the detection of H_2_O_2_. Morphological and surface study of the 3DGA-AuNPs revealed efficacious formation of 3D-networked assembly, which helps in enhancing conductivity and effective enzyme immobilization. The large surface of 3DGA and biocompatibility of AuNPs help in enabling direct electron transfer between the electrode and Cyt c. The as-prepared 3DGA-AuNPs/Cyt c/GCE exhibited a pair of well-defined redox peaks of a Fe^III/II^ redox couple of Cyt c and revealed excellent electrocatalytic potential toward H_2_O_2_ with high sensitivity of 351.57 μA mM^−1^ cm^−2^ [302].

### 3.5. Carbon Nanotubes (CNTs)

CNTs, an allotropic form of carbon, are composed of a graphene sheet packed in a tube constituting a cylinder (single-walled CNTs (SWCNTs)) or concentric and closed tubules (multiwalled CNTs (MWCNTs)) [308,309,310]. The combination of CNTs in biosensors offers numerous advantages, including increased surface area, smooth charge transfer, stacking of various biomolecules, and improved conductivity of the resulting platform as shown in Table 5 [310,311,312].

#### 3.5.1. H_2_O_2_ Electrochemical Sensors Based on the Association of CNTs and Hemoproteins

**Figure 6:**
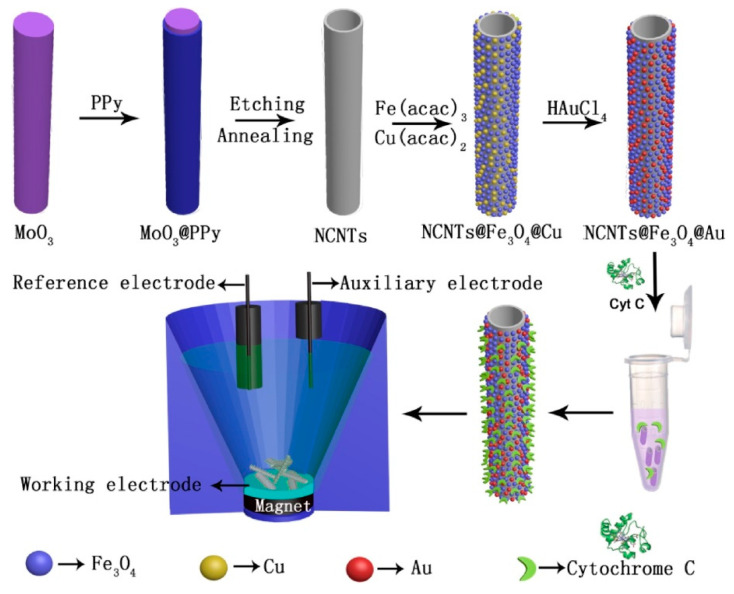
Enzyme-loaded CNTs for the detection of H_2_O_2_ in living cells. Reproduced with permission from [336]. Copyright 2019, Elsevier Ltd (Amsterdam, The Netherlands).

Direct electrochemical assignment of proteins in biosensors is an area of high interest. However, direct electron transfer between proteins and electrodes is faced with major problems, i.e., the distance between the redox center and electrode and protein denaturation. Different methods, including polymer adsorption, covalent binding, and layer-by-layer film assembly, are well known for the deposition of protein molecules on the electrode surface. The excellent electrocatalytic properties of CNTs make them valuable in loading biomolecules and for use as biosensors. CNTs function as nanowires and boost the electron transfer from the protein’s redox center to the electrode. The heme-containing proteins (Hb, Mb, Cyt c, and HRP) are the most common analytes for protein detection [313]. Heme proteins are the center of several biological redox reactions. Therefore, several studies reported the efficacy of these proteins as a biosensor for H_2_O_2_, nitrite, or hydrogen sulfide detection. Yang et al. have developed a method to directly bind hemoglobin to a vertically aligned CNT surface. They modified the nanotubes so they could use diazonium chemistry to directly bind hemoglobin. In amperometric detection of H_2_O_2_, an Hb-ACNT electrode exhibits a wide concentration range (40 μM to 3 mM), LOD of 5.4 M, high sensitivity, and long-term stability [314]. This aligned NT forest shows accumulation of Hb on a large area rather than immobilization in unsystematic tangled webs of CNT. Furthermore, Esplandiu et al. immobilized Mb to detect H_2_O_2_, studied direct electron transfer kinetic, and showed that vertically aligned NT forests possess better kinetics compared to the epoxy incorporated SWCNT/Mb sensor [315]. In addition, their LOD was 50 nM for H_2_O_2_, superior to other random and aligned NT methods. The release of H_2_O_2_ from living HepG2 cancer cells was studied by Zhang and coworkers, who constructed an enzyme-based biosensor with a LOD of 0.23 μM using SWCNTs as a robust scaffold for Hb immobilization. The constructed biosensor was also used for the quantification of H_2_O_2_ released from HepG2 cells via in situ biosynthesis of ZnO quantum dots, which was further confirmed by fluorescence staining [316]. Wang et al. applied a simple dispersion method to coat a GCEs with SWCNT and heme proteins in the presence of cetylrimethylammonium bromide (CTAB) [317]. CTAB-suspended NTs facilitate the immobilization of Mb, Cyt c, and HRP on the electrode surface. Redox chemistry of heme was studied in the presence of SWCNTs. The developed electrode was precisely used for nitrite and H_2_O_2_ detection, which gave rise to a new-fangled peak in cyclic voltammograms with decreased in heme reduction peak. The results indicate that the electrode exhibited a response time of only 4 s, LOD of 3.6 M, and less sensitivity for H_2_O_2_ detection. Several researchers have also demonstrated the efficacy of the immobilization of heme proteins on polymers. For instance, Hb was immobilized on polyelectrolyte surfactant polymers [318], where Hb retained a secondary structure, thus reducing the effect of protein denaturation in polymers. Moreover, the addition of SWCNT to the nanocomposite enhanced the reaction kinetics, and H_2_O_2_ was sensed with a LOD of 0.8 M. Likewise, MWCNT and Mb were immobilized on the collagen polymers [319], where H_2_O_2_ was measured with a linear range from 0.6 to 39 M. Nagaraju et al. used self-assembled monolayers of 4-aminothiophenol on gold electrodes with immobilized Cyt c for H_2_O_2_ detection [320]. Three orders of magnitude of faster electron transfer kinetics were observed with SWCNT in these monolayers as compared to the non-SWCNT monolayer. The results confirmed that NTs increased the direct electron transfer. However, large step changes (3.8 mM) in H_2_O_2_ were used, and no LOD was calculated. Thus, the sensitivity was not very significant. A layer-by-layer approach was also implemented to immobilize proteins rather than simultaneous deposition of all components, e.g., chitosan-stabilized NTs were placed on GCE, followed by the accumulation of gold NPs on chitosan, and subsequently, Hb was bound to the gold surface [321]. This method is beneficial in retaining the bioactivity of Hb and increases the amount of enzyme activity. The method showed a LOD of 0.2 M for H_2_O_2_ detection. The heme-based biosensors showed rapid and fast detection of changes in H_2_O_2_. In general, HRP is the most widely used in biosensing as compared to other heme-containing proteins, which shows the best results compared to others. The lowest limits of detection for H_2_O_2_ were achieved using an aligned NT geometry, which supports the accumulation of the heme protein and which, in turn, leads to fast electron transfer from proteins to electrodes. Future studies are required to address the reproducibility of electrode fabrication, practical geometries, and uses for real samples. SWCNTs, HRP, and 1-butyl-3-methylimidazolium tetrafluoroborate (BMIM·BF_4_) were combined to construct a cellular H_2_O_2_ sensor. At a working potential of −0.35V, HRP-BMIM·BF4/SWCNTs/CFUME showed a dynamic range of ~10.2 μM, with a low detection limit of 0.13 μM (*S*/*N* = 3) and high sensitivity of 4.25 A/M cm^2^. Due to its small dimension and low working potential, HRP-BMIM·BF_4_/SWCNTs/CFUME allowed direct amperometric real-time monitoring of H_2_O_2_ in HeLa cells treated with camptothecin (an anticancer drug) without complex data processing and extra surface coatings to prevent interference. Thus, its testing evidently demonstrated a significantly high level of H_2_O_2_ in HeLa cells under camptothecin stress [322]. A schematic presentation of enzyme loaded CNTs for the detection of H_2_O_2_ in living cells is shown in Figure 6.

**Table 5:** CNTs-supported metal nanoparticles biosensors for electrochemical sensing of H_2_O_2_ secreted from cancerous cells.

**CNTs H_2_O_2_ Biosensors**	Sensitivity μA mM^−1^ cm^−2^	Linear Range (μM)	Detection Limit (μM)	Ref.
ZnO/COOH-MWNTs	-	1–21	--	[323]
GCE/MWCNTs-CDs	0.039	3.5–300	0.25	[324]
((APy)_6_[H_2_W_12_O_40_])/(SWCNT-COOH)	-	-	0.4	[325]
GCE/CNTs-PAMAM DENs-PtNCs	987.5	3–400	0.8	[326]
GCE/C_60_-MWCNTs CS-IL/MB/CuNP	0.0243	2–4	0.055	[327]
3D PB NPs/G-CNTs	0.11343	1–3161	0.095	[328]
CF@N-CNTAs–AuNPs	142	1–4300	0.05	[329]
CDs/MWCNTs/GCE	--	--	-	[324]
OECT/PET/CE-CNTs/PtNPs	-	0.5–100	0.2	[330]
ZNBs/fMWCNTs	-	0.049–22	0.035	[331]
ZnONPs/MWCNTs	-	1000–200,000	-	[332]
N-CNTs	30	-	0.5	[333]
(GC) (BG-CNPs/GC)	-	-		[334]
GCE/rGONRs/MnO_2_	0.0142	0.25–2245	0.071	[335]

#### 3.5.2. H_2_O_2_ Electrochemical Sensors Based on the Association of Metallic Nanoparticles and CNTs

**Figure 7:**
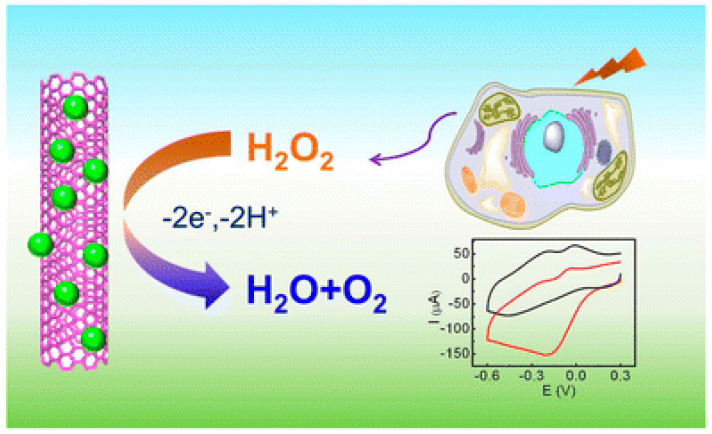
Mechanism of metal nanoparticle loaded CNTs for real-time analyses of H_2_O_2_ secreted from live cells. Reproduced with permission from [324], Copyright 2016 Spinger Ltd (Berlin/Heidelberg, Germany).

In the last few years, the connotation of metal nanoparticles with CNTs has been considered a valuable alternate for the development of highly sensitive and selective sensors for H_2_O_2_ detection shown in Figure 7 [41,337,338]. Zhang et al. developed a remarkable stretchy nanohybrid microelectrode using carbon fibers [329]. The constructed microelectrode reproduced a remarkable analytical signal at 0.300 V, with an ultrasmall LOD as low as nanomolar. Rapid and ultrasmall sensing of H_2_O_2_ excreted from MCF-7 and MDA-MB-231 cells was achieved because of the synergistic catalytic activity of the N-CNTA-decorated AuNPs. Table 6 showed non-enzymatic CNTs biosensors for H_2_O_2_ sensing.

Bai et al. developed an electrode with high electrocatalytic activity using carbon dots (CDs) and oxidized MWCNTs modified GCEs [324]. The excellent biosensing capability of the MWCNT/CD/GCEs composite is associated with the large surface area and electron acceptor characteristics of MWCNTs and with the excellent donor capacity of the CDs. The analytical performance of the sensor is highly dependent on the MWCNT:CD ratio, so 10:1 was selected for electrode manufacturing. The developed biosensors successfully quantified the H_2_O_2_ secreted from HeLa cells with a linear range of 3.5 × 10^−6^ and 3 × 10^−4^ M and a LOD of 0.25 mM. 

Liu and Ding used a Pt-encapsulated poly(amidoamine) dendrimer with amine terminations (G6-NH_2_ PAMAM dendrimer) covalently attached to a carboxylated CNT composite [326]. Elaborated architectures showed a rapid, reproducible, and steady response at 0.150 V, with a linear range between 3 and 400 mM. The biosensor successfully detected H_2_O_2_ in MCF-7 cells with an LOD of 0.8 mM. Liu at al. modified GCEs by dendrimer-encapsulated Pt nanoclusters and carbon nanotubes (Pt-DENs/CNTs) to detect extracellular H_2_O_2_ excreted from live cells. Those Pt-DENs/CNTs nanocomposites were characterized by UV-Vis spectra, SEM, energy-dispersive X-ray spectroscopy, and TEM. The nonenzymatic sensor displayed exceptional catalytic activity in H_2_O_2_ reduction. The effective nonenzymatic sensing capability of H_2_O_2_ reduction revealed that the Pt-DENs/CNTs sensor has potential application in screening H_2_O_2_ in cellular processes [326].

Real-time monitoring of H_2_O_2_ secreted from living cells is important to understand the occurrence of diseases and searching of new therapeutic strategies. Zhao et al. successfully synthesized three-dimensional carbon nanotubes spaced graphene aerogel decorated with Prussian blue nanoparticles (3D PB NPs/G-CNTs) by one-step mild temperature treatment, in which the PB NPs acquire intrinsic peroxidase-like activity. The 3D porous structure of G-CNTs with large surface and high electrical conductivity can efficiently enhance catalytic performance and help in real-time detection of H_2_O_2_ released from living cells. The composite exhibited good catalytic performance toward H_2_O_2_ reduction with sensitivity of 134.3 μA mM^−1^ cm^−2^, LOD of 95 nM, and wide linear range of 1–3161 μM [328]. 

Zhang et al. integrated Fe_3_O_4_ and Cu nanoparticles (NPs) into the NCNTs to produce N-doped carbon@Fe_3_O_4_-Cu nanotubes (NCNTs@Fe_3_O_4_@Cu) through a one-pot high-temperature decomposition. Then, Au NPs were assembled on the magnetic NCNTs to obtain an NCNTs@Fe_3_O_4_@Au composite by galvanic replacement with Cu NPs. The resultant composite provided a suitable platform for the immobilization of the enzyme to fabricate biosensors for H_2_O_2_ monitoring. After the cytochrome c (Cyt c) was accumulated by the NCNTs@Fe_3_O_4_@Au composite, the Cyt c/NCNTs@Fe_3_O_4_@Au gathered to the surface of the electrode with an external magnet [336]. Tabrizi et al. developed a flow injection amperometric sandwich-type aptasensor for the detection of human leukemic lymphoblasts (CCRF-CEM). An amperometric biosensor was synthesized by decorating nanogold on poly(3,4-ethylenedioxythiophene) (PEDOT-Au_nano_). PEDOT-Au_nano_ acts as a nano-platform for immobilizing a thiolated sgc8c aptamer and MWCNTs loaded PdNPs/3,4,9,10-perylene tetracarboxylic acid (MWCNTs-Pd_nano_/PTCA) to assemble a catalytic labeled aptamer. In this strategy, the CCRF-CEM cancer cells were sandwiched between the immobilized sgc8c aptamer on PEDOT-Au_nano_ (electrode) and sgc8c aptamer MWCNTs-Pd_nano_/PTCA/aptamer (catalytic site). The resultant sandwich-type aptasensor determined the CCRF-CEM cancer cell concentration using 0.1 mM H_2_O_2_ (electroactive component). The MWCNTs-Pd_nano_ nanocomposites enhanced the electrocatalytic reduction of H_2_O_2_, which further boost sensor sensitivity toward CCRF-CEM cancer cells. The proposed sandwich-type aptasensor displayed outstanding analytical performance for real-time determination of CCRF-CEM cancer cells with high selectivity, ranging from 1.0 × 10^1^ to 5.0 × 10^5^ cells mL^−1^ with an LOD of 8 cells mL^−1^ [339].

#### 3.5.3. In Vivo Sensing of H_2_O_2_ Release from Carcinoma Cells

Considering the significance of cellular H_2_O_2_ in cell pharmacology and pathophysiology, accurate and reliable in vivo detection of cellular H_2_O_2_ is sorely needed. Sensing H_2_O_2_ at cellular level is constrained by several factors, including small cell size, low concentration of cellular H_2_O_2_, and interferences in the culture medium [215,340]. Such in situ monitoring of the cellular release of H_2_O_2_ provides a new in vitro drug screening platform for personalized medicine and cancer therapy. Enzyme-based electrochemical sensing is efficient for continuous in situ monitoring of H_2_O_2_ because of its high sensitivity, rapid response, and selectivity [27,267]. In vivo monitoring of H_2_O_2_ secreted from living cells is essential in understanding cellular signaling pathways. The release of H_2_O_2_ from living cells is very low because the selective detection of H_2_O_2_ at a low level is challenging. 

Chen et al. used a flow-through mode sensing strategy based on cell-in-lumen configuration for ultra-small detection of H_2_O_2_ secreted from the H1299 carcinoma cell. The current strategy involved the growth of cells on the inner surface of a porous hollow fiber (PHF), while a sensing layer comprised of multi-walled carbon nanotubes, gold nanoparticles (AuNPs), and enzymes accumulated on the outer surface of the PHF. The porous structure of the resultant electrode proved beneficial in the exchange of H_2_O_2_ from cell to sensing layer in a short time span. The resultant electrode exhibits ultra-small sensitivity to detect H_2_O_2_ at the nanomolar level having a detection limit of 6 nM with a wide linear range of 0.01–5 [341]. Ye et al. fabricated a PdPt NCs@SGN/GCE non enzymatic electrochemical biosensor comprised of Pd-Pt nanocages and SnO_2_/graphene nanosheets. The resultant electrode displayed excellent catalytic activity toward H_2_O_2_ in situ secreted from human cervical cancer cells (Hela cells) with high selectivity and sensitivity, a low detection limit of 0.3 mM, and a large linear range from 1 mM to 300 mM [303]. Fe_3_O_4_ quantum dot was decorated on three-dimensional graphene nanocomposites (Fe_3_O_4_/3DG NCs) for real time in-situ monitoring of H_2_O_2_ released from living cancer cells. The fabricated electrochemical sensor mimics peroxidase-like activity with high sensitivity of 274.15 mA M^−1^ cm^−2^, a low detection limit (78 nM), fast response (2.8 s), and outstanding reproducibility [342]. 

A nonenzymatic electrochemical sensor was constructed by immobilizing 2D ultrathin MnO_2_ nanosheets onto glassy carbon electrodes (GCE) with a Nafion film for real-time monitoring of H_2_O_2_ released from SP2/0 cells in trace amounts. The amperometric study showed an excellent increase in electrocatalytic reduction of H_2_O_2_ with an extreme low detection limit (5 nM), wide linear range (25 nM^−2^ μM and 10–454 μM), and high sensitivity of 3261 mA M^−1^ cm^−2^ via the immobilization of the MnO_2_ nanosheets [239]. Xi et al. synthesized N and S dual-doped graphene (NSG) co-doped carbocatalyst via one-pot syntheses. The NSG-modified electrode showed superior catalytic activity toward sensing, including a linear range up to 1.7 mM. The prepared electrode showed high sensitivity of 0.266 mA cm^−2^ mM^−1^ with a detection limit as low as 1 μM (*S*/*N* = 3), with good discernment, reproducibility, stability, and biocompatibility with real-time determination of H_2_O_2_ secreted from live cancerous cells [343]. Later on, Zhao et al. used a well-controlled strategy for the syntheses of the graphene fiber microelectrode via MnO_2_ nanowire (MnO_2_-NWs) assembly (MnO_2_-NWs@Au-NPs). The prepared microelectrode showed proficient catalytic performance toward the redox reaction of H_2_O_2_. The nanohybrid microelectrode showed in vivo real-time detection of H_2_O_2_ released from human breast cancer cells [344]. Recently, Chen and his co-worker established high-index facets of an Au-Pd nanocubes loaded rGO composite. The resultant electrode comprised of three-dimensional nanocomposites showed a detection limit of 4 nM, a wide linear range from 0.005 μM to 3.5 mM, and real time monitoring of endogenous H_2_O_2_ in human serum samples released from a living breast cancer cell [345]. 

### 3.6. MXenes Materials

So far, various nanomaterials have been reported and utilized for the development of incrementally efficient biosensors. Among the most recently reported nanomaterials available for biosensors, MXenes have attracted much attention for their huge potential in biosensor development because of their unique characteristics [346]. MXenes are two-dimensional inorganic compounds with a thickness of a few atomic layers and they are composed of transition metal carbides, nitrides, or carbonitrides such as titanium carbide (Ti_3_C_2_) and titanium carbonitride (Ti_2_CN), which confers them with exceptional characteristics, including high conductivity and superior fluorescent, optical, and plasmonic properties [347,348]. Moreover, the biocompatible property of MXenes enables their biomedical application [349,350]. Since they were first reported in 2011, MXenes have been used to develop various types of advanced biosensors, including electrochemical, fluorescent/optical, and surface-enhanced Raman spectroscopy (SERS) biosensors, by augmenting MXene characteristics to make them suitable for specific types of biosensors or by combining them with other nanomaterials [351,352]. Recent studies on the development of highly effective MXene biosensors show that this novel nanomaterial is the most ideal candidate for biosensor development at present. So far, no considerable development was seen in MXenes-based biosensors for detection of H_2_O_2_ released from a cancer cell. However, we foresee MXenes as having outstanding potential for detection of H_2_O_2_ at an ultra-low level with durable stability and long working hours.

## 4. Conclusions and Future Perspectives

Carbon nanomaterials have gained prodigious attention over the last two decades because of their higher applicability in electrochemical sensors. This review shed light on the application of carbon nanomaterials and their composite with metal, metal oxides, and biomolecules for the fabrication of electrochemical sensors for real-time monitoring of hydrogen peroxide. Initially, we discussed the recent advancement in the development of heme protein biosensors with carbon nanomaterials as immobilization matrix and their application in the detection of H_2_O_2_. Subsequently, the synthesis and application of graphene-supported nano-catalysts (metal-free, noble metals, and nonnoble metals) was discussed in detail for the construction of nonenzymatic H_2_O_2_ electrochemical sensors. Despite the extensive advancement in the design and application of carbon nanomaterials for the electrocatalytic determination of H_2_O_2_, it is crucially important to develop new techniques and methods for the synthesis of carbon-based electrocatalysts with a novel structure and extraordinary activity. Some of the most highly ultra-sensitive biosensors for detecting H_2_O_2_ at an ultra-low level are displayed in Table 6. Furthermore, the comprehensive understanding and exploration of the structure–property relationship of carbon nanomaterials and their extensive use in H_2_O_2_ sensors require more efforts and research. Particularly, its excellent electrical conductivity, electron mobility, small band gap, and ultrahigh surface area make it widely applicable in biosensors. These advantages of graphene would bestow good conductivity to the capsule film, further facilitate fast electron transfer between enzyme and basal electrode, and enhance the sensitivity and detection limit of biosensors. We prophesy excellent biosensing potential of new MXenes materials and carbon-based material for detection of H_2_O_2_ released from cancer cells at an ultra-low level with remarkable stability and selectivity.

## Figures and Tables

**Table 1:** Non-enzymatic metal free H_2_O_2_ electrochemical sensors based on graphene.

Carbon Material	Sensitivity μA mM^−1^ cm^−2^	Liner Range (μM)	Detection Limit (μM)	Ref.
CR-GO	-	0.05–1500	0.05	[155]
Graphene-MWCNT	32.91	20–2100	9.4	[196]
rGO/nPPy	47.69	0.1–4	0.034	[201]
IL-GR-s-PANI	280.0	0.5–2000	0.06	[202]
rGO/Tyrosine	69.07	100–2100	80	[203]
Poly(o-Phenylenediamine)/GO	16.2	2.5–25	0.84	[204]
BGNs	266.7	1000–20,000	3.8	[199]
NB-G	-	0.5–5000	0.05	[200]
GSnano/CS	18.78	5.22–10,430	2.6	[205]
GN-HN-SWCNT	0.015	0.2–400	0.05	[206]
H-GNs/PEDOT	235	0.1–10	0.08	[207]
NS-GQD/G	-	0.4–33	0.026	[208]
Functionalized 3D Graphene	169.7	0.4–660	0.08	[209]
rGO/GO hybrid MEA	-	0.18–9.6	-	[210]
3D-G/GCE	-	0.2–41,200	0.17	[211]

**Table 2:** Graphene-based enzymatic biosensors for H_2_O_2_ detection in Cancerous cells.

**Graphene-Based Materials**	**Sensitivity** μA mM^−1^ cm^−2^	**Linear Range** **(** **μ** **M)**	**Detection Limit** **(** **μ** **M)**	**Ref.**
GE/Fe_3_O_4_/Hb GCE	0.3837	100–1700	6.00	[218]
rGO-CMC/Hb	-	0.083–13.94	0.08	[219]
Hb/AuNPs/ZnO/Gr	-	6.0–1130	0.8	[220]
HRP/graphene	-	0.33–14.0	0.11	[221]
Hb/Au/GR-CS	3.47 × 10^5^	2.0–935	0.35	[222]
Hb/Au NPs-Gr	-	0.1–70	0.03	[223]
HRP/P-L-His-rGO	2.6 × 10^5^	0.2–5000	0.05	[224]
GS-PSS/GRCAPS	-	10–12,000	3.3	[216]
HRP/AuNP/ThGP	0.086	0.5–1800	0.01	[225]
PANI/HRP/GE-CNT/AuPt NPs	370	0.5–100	0.17	[226]
Au/graphene/HRP/CS	-	5.0–5130	1.7	[227]
MP11/DMPG-AuNPs/PDDA-G	243.7	20–280	2.6	[228]
(HRP-Pd)/f-graphene	92.82	25–3500	0.05	[229]
HRP-f-graphene-Ag	143.5	25–19,350	5.0	[230]
HRP/CeO_2_-rGO	4.65	0.1–500	0.021	[212]
HRP-MoS_2_-Gr	679.7	0.2–1103	0.049	[214]
Catalase/AuNPs/graphene-NH_2_	13.4	0.3–600	0.05	[231]
Cyt c/GO-MWCNT/Au NP	0.533	1 × 10^−^^5^–1.4 × 10^−4^	27.7 × 10^−6^	[232]
RGO-MWCNT-Pt/Mb	1.990	1 × 10^−5^–1.9 × 10^−4^	16 × 10^−6^	[233]
PPY-He-RGO	-	0.1–10	0.13	[41]
HRP/PGN/GCE	-	8.0 ×10^−11^–6.64 × 10^−7^	2.6 × 10^−5^	[215]
PGR/catalase/GCE	-	1.0 × 10^−7^–7.7 × 10^−6^	1.5 × 10^−3^	[234]
FeS_*x*_/graphene	-	-	5 × 10^−4^	[235]
F-MoS_2_-FePt NCs	-	8–300	2.24	[236]

**Table 3:** Graphene-supported non-Noble metal nanoparticles for electrochemical detection of H_2_O_2_.

Graphene-Based Materials	Sensitivity μA mM^−1^ cm^−2^	Linear Range (μM)	Detection Limit (μM)	Ref.
Nafion/EGO/Co_3_O_4_	560	1–100	0.3	[240]
CoHCFNPs/GR	0.0007	0.6–379.5	0.1	[41]
VS_2_ NPs/GCE	41.96 2	0.5–2.5	0.224	[241]
CoO*_x_*NPs/ERGO	148.6	5–1000	0.2	[242]
CoTPP/RGO	0.0013	0.1–4600	0.02	[243]
rGO/CoPc-COOH	14.5	100–12,000	60	[244]
(PDDA-G/Fe_3_O_4_)n	61.2	20–6250	2.5	[245]
Fe_3_O_4_/GO-PAMAM	1.385	20–1000	2.0	[246]
CoS/RGO	2.519	0.1 to 2542.4	0.042	[247]
rGO-Fe_2_O_3_	0.085	50–9000	6.0	[248]
Fe_3_O_4_/rGO	387.6	1–20,000	0.17	[249]
Ni_2_P NA/TM	690.7	0.001–20	0.2	[166]
Fe_3_O_4_/RGO	22.27	0.5–3000	0.18	[41]
Fe_3_O_4_/RGO	0.0468	4.0–1000	2.0	[250]
PB/TiO_2_-GR	480.97	0.04–2000	0.0086	[251]
RGO/Fe_3_O_4_	688.0	100–6000	3.2	[252]
Cu-MOF-GN	57.73	10–11,180	2.0	[33]
PFECS/rGO	117.142	10–190	1.253	[253]
FeTSPc-GR-Nafion	36.93	0.2–5000	0.08	[254]
RGO/ZnO	13.49	0.02–22.48	0.02	[255]
Cu_2_O/N-graphene	26.67	5.0–3570	0.8	[256]
Cu_2_O-rGO	0.0207	30–12,800	21.7	[257]
CuS/RGO	0.035	5–1500	0.27	[80]
Cu_2_O/GNs	-	300–7800	20.8	[258]
GO/MnO_2_	38.2	5.0–600	0.8	[259]
MnO_2_-ERGO	59.0	100–45,400	10	[260]
MnO_2_ nanosheet/graphene	-	10–900	2.0	[261]
CoFe/NGR	435.7	-	0.28	[262]
Co@NCS	-	0.5–7500	0.08	[263]

**Table 6:** Ultra-sensitive electrochemical biosensors for detection of H_2_O_2_.

**H_2_O_2_ Biosensors**	Sensitivity μA mM^−1^ cm^−2^	Linear Range (μM)	Detection Limit (μM)	Ref.
GN-HN-SWCNT	0.015	0.2–400	0.05	[206]
Cyt c/GO-MWCNT/Au NP	0.533	1 × 10^−^^5^–1.4 × 10^−4^	27.7 × 10^−6^	[232]
HRP/P-L-His-rGO	2.6 × 10^5^	0.2–5000	0.05	[224]
HRP/AuNP/ThGP	0.086	0.5–1800	0.01	[225]
RGO-MWCNT-Pt/Mb	1.990	1 × 10^−5^–1.9 × 10^−4^	16 × 10^−6^	[233]
CoTPP/RGO	0.0013	0.1–4600	0.02	[243]
GN-Pt	0.01	2–710	0.5	[277]
PDA-RGO/Ag NP	0.0111	0.5–8000	2.07	[282]
Pt/GN	0.0204	2.5–6650	0.8	[41]
PtAuNPs-CTAB-GR	0.1654	0.005–4.8	0.0017	[294]
Pd-NPs/GN	0.019	0.001–2000	0.0002	[298]
GCE/MWCNTs-CDs	0.039	3.5–300	0.25	[324]
GCE/C_60_-MWCNTs CS-IL/MB/CuNP	0.0243	2–4	0.055	[327]
GCE/rGONRs/MnO_2_	0.0142	0.25–2245	0.071	[335]

## Data Availability

All data are available from the corresponding author upon request.
